# Climate-induced shifts in leaf unfolding and frost risk of European trees and shrubs

**DOI:** 10.1038/s41598-018-27893-1

**Published:** 2018-06-29

**Authors:** Christof Bigler, Harald Bugmann

**Affiliations:** Forest Ecology, Institute of Terrestrial Ecosystems, Department of Environmental Systems Science, ETH Zurich, Zurich, Switzerland

## Abstract

Climate warming has advanced leaf unfolding of trees and shrubs, thus extending the growing period but potentially exposing plants to increased frost risk. The relative shifts in the timing of leaf unfolding *vs*. the timing and intensity of frost events determine whether frost risk changes under climate warming. Here we test whether the frost risk for unfolding leaves of 13 European tree and shrub species has changed over more than 60 years using dynamic state-space models and phenological observations from 264 sites located between 200 and 1900 m a.s.l. across Switzerland. Trees and shrubs currently feature sufficient safety margins regarding frost risk, which increase from early- to late-leafing species and tend to decrease with increasing elevation. Particularly after 1970 to 1990 and at higher elevations, leaf unfolding has advanced across all species. While the time between the last critical frost and leaf unfolding has shifted from predominantly positive trends in the late 1950s and 1960s to a trend reversal since the 2000s, the minimum temperature during leaf unfolding has mostly increased since the 1980s. These dynamic shifts in leaf unfolding and frost risk demonstrate species- and site-specific responses of trees and shrubs to climate cooling and warming.

## Introduction

Plants have evolved phenological adaptations in response to seasonal weather variation in temperate climates^1^. Perennial plants undergo a period of cold acclimation in fall by increasing frost resistance, followed by dormancy in winter when maximum frost resistance is reached, and deacclimation in spring concurrent with decreasing frost resistance^1–3^. The timing of spring phenophases such as leaf unfolding or flowering is determined by an inverse relationship between accumulated chilling temperatures (i.e., cool temperatures in winter that break dormancy) and forcing temperatures (i.e. high temperatures in spring following dormancy that promote cell growth)^1,4^. Photoperiod may compensate for a lack of sufficient chilling in some species^1,4^. Recent shifts in the timing of leaf unfolding and flowering reflect one of the most evident biotic responses to global warming^5,6^. These climate-induced changes of plant phenology affect species distributions^7,8^ and the seasonal cycling of terrestrial and atmospheric carbon, water and nutrients^9–11^.

**Figure 1:**
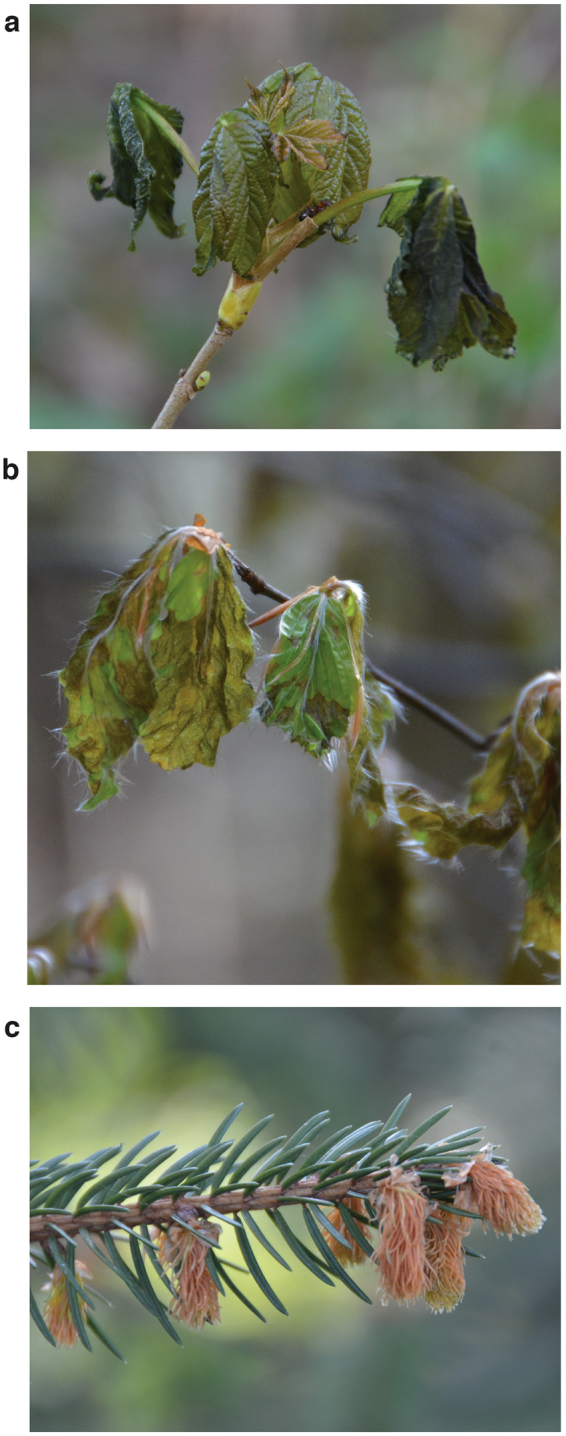
Frost damage on unfolding leaves following late spring frost. Pictures of leaves from (**a**) sycamore (*Acer pseudoplatanus*), (**b**) European beech (*Fagus sylvatica*) and (**c**) Norway spruce (*Picea abies*) were taken on 24 April 2017 (Zugerberg, Switzerland; ca. 47.14°N, 8.53°E; sycamore and beech at 940 m a.s.l., spruce at 800 m a.s.l.). Spring temperatures based on daily average temperatures (T_ave_) from 16 March to 5 May 2017 were 1.18 K above the long-term mean (2017: 6.42 °C; 1931 to 2016: 5.24 ± 1.43 °C, mean ± sd) and resulted in unusually early leaf unfolding. The warm spring was followed by two severe frost events (daily minimum temperatures T_min_ of −6.8 °C during the nights from 19–20 and 20–21 April 2017). Climate data are from the nearby station Einsiedeln (ca. 47.13°N, 8.76°E, 910 m a.s.l.). Photographs by C. Bigler.

Leaf unfolding has advanced for the majority of tree and shrub species in response to increasing spring temperatures^5,10,12^. While early leaf unfolding allows a tree or shrub to extend the growing period and thus to produce more assimilates and increase biomass production, it also entails an increased risk of frost damage^13^. Since frost resistance is low during leaf unfolding, plants are particularly vulnerable to frost damage when temperatures drop below species-specific critical values (Fig. 1). Frost damages in leaves occur due to the formation of intra- or extracellular ice crystals that induce rupture of biomembranes and cellular dehydration^2,8^, which eventually result in partial or total loss of plant leaf area and a reduction in stored carbon and nutrients^11,14^.

Climate warming is not only reflected in increasing spring temperatures that drive leaf unfolding, but it also increases the variability of frost events through changes in the seasonal distribution and intensity of frost events^15^. The observed rate of warming tends to increase with elevation, particularly for minimum temperatures^16^. Thus, the relative temporal shifts in the timing of leaf unfolding and frost events at a given elevation determine whether frost risk will change under climate change^17,18^. Previous studies have evaluated whether the frost risk for leaf phenology in trees and shrubs changes (1) between current and future climate conditions using simulated leaf phenology and weather data^17,19^; (2) between ambient and elevated air temperature using whole-tree *in-situ* experiments^20,21^; (3) between low- and high-elevation sites^22^ or along elevational transects^23^ using observational data; and (4) over time, typically using linear models and without considering species-specific frost resistance^18,24–26^. There is little consensus whether frost risk increases or decreases under global warming (for an overview see refs^1,2,13^), which likely reflects the fact that previous studies considered relatively few species, sites, or elevations. To date, no comprehensive study has empirically assessed whether frost risk during leaf unfolding (i) differs across species that cover a wide spectrum of leaf unfolding dates and frost resistances, (ii) changes along extensive elevation and thus temperature gradients, and (iii) reflects time-varying trends over longer periods.

**Figure 2:**
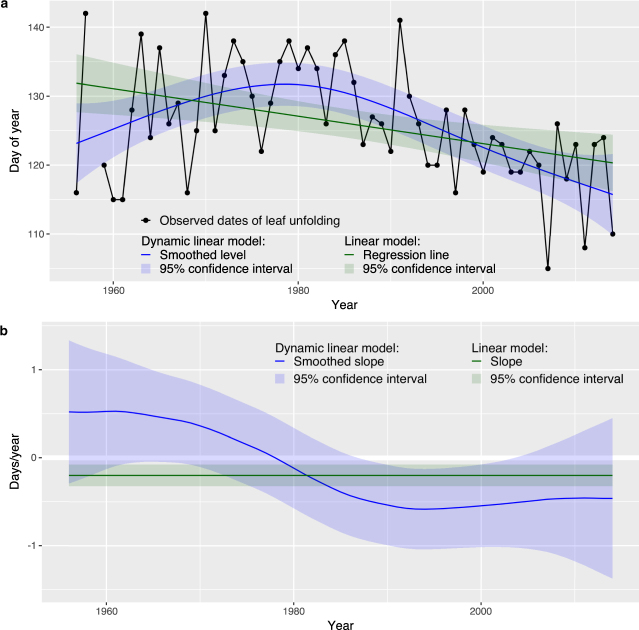
Trend analysis of leaf unfolding based on dynamic linear model (DLM) and linear model (LM). Data for beech at the station La Valsainte (see Supplementary Table S1 and Supplementary Fig. S9): (**a**) observed dates of leaf unfolding, smoothed levels based on DLM, and regression line based on LM, (**b**) smoothed slopes based on DLM and slope based on LM. The DLM indicates a positive slope in 1960 (0.53 days/year) and a negative slope in 2010 (−0.46 days/year), whereas the LM suggests a constant negative slope (−0.20 days/year).

Here, we tested over more than 60 years whether frost risk during leaf unfolding of European tree and shrub species has changed. We analyzed nearly 48’000 dates of observed leaf unfolding from 13 deciduous and conifer species that include a gradient from early- to late-leafing species (see Methods). The phenological observations were recorded from 1951 to 2014 at 264 phenological stations located between 200 and 1900 m a.s.l. across Switzerland, thus widely different environmental conditions from sub-mediterranean to subalpine are covered. We combined these leaf unfolding data with interpolated daily weather data of minimum temperature (T_min_) and average temperature (T_ave_). To investigate overall frost trends across Switzerland, we extracted the last spring frosts based on fixed temperature thresholds (T_min_ < −1 °C, …, <−10 °C). To investigate trends of frost risk during leaf unfolding, we considered species-specific estimates of frost resistance (LT_50_, lethal temperature for 50% of the unfolding leaves) and calculated the (1) date safety margin^22^ (difference between date of leaf unfolding and date of last spring frost with T_min_ < LT_50_) and (2) temperature safety margin^22^ (difference between lowest T_min_ within 5 days before and after leaf unfolding and LT_50_). We analyzed trends using dynamic linear models (DLMs; see Methods), which represent a little explored method for stochastic trend detection from the broad class of state-space models^27^. DLMs allow for the modeling of time-varying trends (Fig. 2), unlike commonly used linear models (LMs).

## Results and Discussion

**Figure 3:**
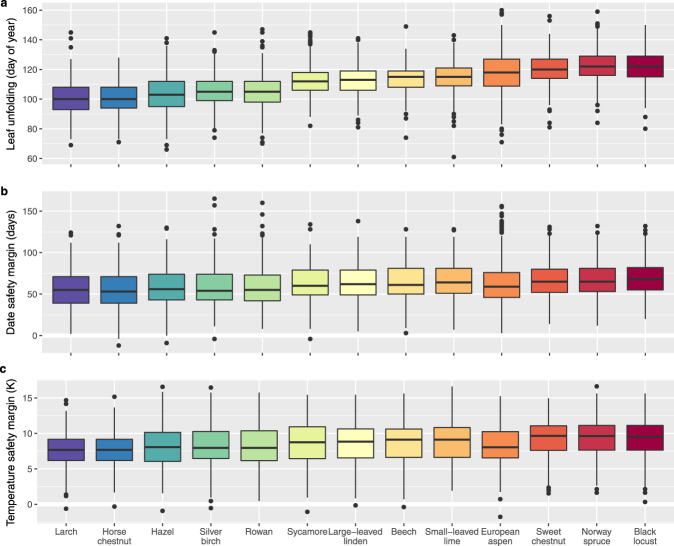
Distributions of leaf unfolding dates and frost risk (date safety margin and temperature safety margin). Box plots of: (**a**) leaf unfolding dates, (**b**) date safety margins (equation 2), and (**c**) temperature safety margins (equation 3). Boxes include the 25th, 50th and 75th percentiles. To ensure comparability, only observations for the common period (1996 to 2011; 1980 to 1995 for European aspen) at the 34 common stations are shown (number of observations: leaf unfolding dates, n = 5′827; date safety margins, n = 5′484; temperature safety margins, n = 5′827). The species are ordered from early- (blue) to late-leafing species (red) according to median dates of leaf unfolding. See Supplementary Fig. S3 for leaf unfolding and safety margins at all stations from 1951–2011; see Supplementary Figs S1, S2, and S4 for leaf unfolding and safety margins in different elevation bands.

Leaf unfolding of the earliest species (larch, horse chestnut) occurred ca. 3 weeks before the latest species (Norway spruce, black locust) over the common period 1996 to 2011 and at those 34 stations where all species were observed (Fig. 3a). This pattern was consistent across elevations based on all stations (Supplementary Fig. S1). Leaf unfolding at the highest stations (≥1100 m) was delayed by ca. 9 days for black locust up to ca. 26 days for European aspen and hazel compared to the lowest stations (<500 m) (Supplementary Fig. S1). Across all species, the date safety margins of frost risk ranged from 27 to 109 days (2.5% to 97.5% percentiles), and only 0.09% of the values were negative at the 34 common stations (Fig. 3b; 0.17% for all stations from 1996 to 2011, Supplementary Fig. S2; 0.19% for all stations from 1951 to 2011, Supplementary Fig. S3b). The date safety margins increased from early-leafing species (horse chestnut, 55.9 ± 21.4 days; larch, 56.1 ± 20.2 days; mean ± standard deviation) to late-leafing species (Norway spruce, 67.3 ± 20.7 days; black locust: 69.2 ± 20.5 days; Fig. 3b). Within species, the date safety margins decreased with increasing elevation (Supplementary Fig. S2), which is likely due to the more rapid increase in temperature during leaf unfolding^22,23^. Across all species, the temperature safety margins of frost risk ranged from 3.15 K to 13.64 K (2.5% to 97.5% percentiles), and 0.14% of the temperature safety margins were negative for the 34 common stations (Fig. 3c; 0.24% for all stations from 1996 to 2011, Supplementary Fig. S4; 0.28% for all stations from 1951 to 2011, Supplementary Fig. S3c). The temperature safety margins increased from early-leafing species (larch, 7.67 ± 2.50 K; horse chestnut, 7.83 ± 2.56 K) to late-leafing species (Norway spruce, 9.24 ± 2.78 K; black locust, 9.30 ± 2.60 K; Fig. 3c). Within species, the temperature safety margins remained constant or decreased slightly with increasing elevation (Supplementary Fig. S4), which confirms earlier findings^3,22^.

**Figure 4:**
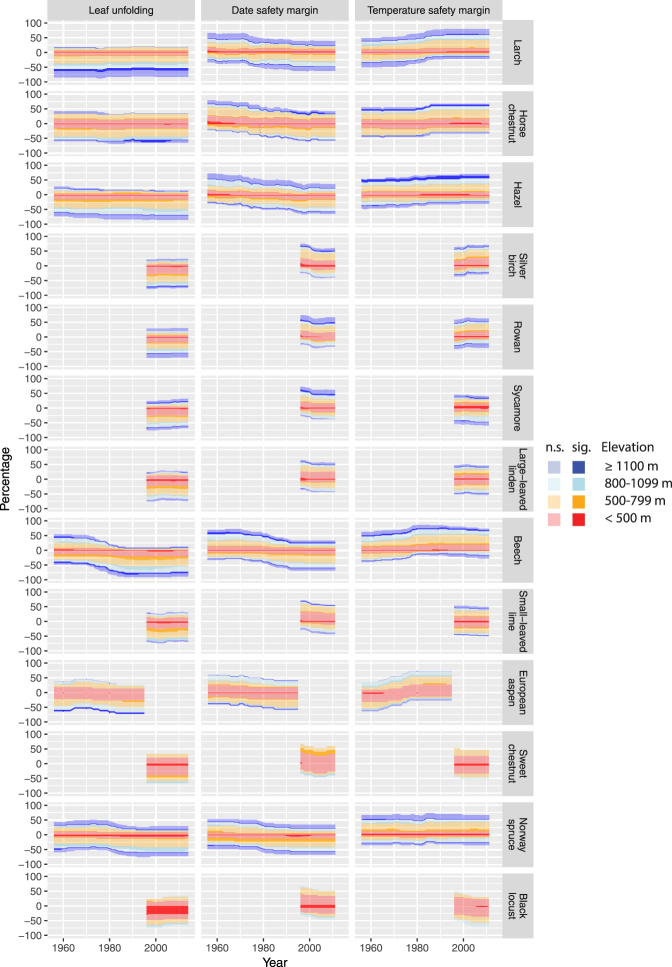
Shifts in leaf unfolding dates and frost risk (date safety margin and temperature safety margin) based on dynamic linear models (DLMs). Shown are percentages of stations assigned to four elevation bands with positive smoothed slopes (positive percentages on y-axis) and negative smoothed slopes (negative percentages on y-axis; see equations 5–8). The species are ordered according to median dates of leaf unfolding (see Fig. 3a). Only series from 1956 (1996 for species with later beginning of observations) to 2011 (2014 for leaf unfolding; 1995 for European aspen) without missing estimates of smoothed slopes are shown (number of series: larch, n = 46–47; horse chestnut, n = 31–33; hazel, n = 39–44; silver birch, n = 74–77; rowan, n = 90–94; sycamore, n = 79; large-leaved linden, n = 70–73; beech, n = 41–42; small-leaved lime, n = 63–64; European aspen, n = 22; sweet chestnut, n = 15; Norway spruce, n = 35–36; black locust, n = 29–30). Non-significant slopes (n.s.) are shown with semi-transparent colors, significant slopes (sig.) with opaque colors. For each species and year, the absolute values of positive and negative percentages across all elevation bands sum up to 100%. See also Supplementary Figs S8, S12 and S13 for observations, smoothed levels and slopes of leaf unfolding and safety margins, Supplementary Fig. S14 for results based on all series, and Supplementary Fig. S7 for results based on linear models (LMs).

The trend analysis using DLMs showed that leaf unfolding has been advancing across all species (Fig. 4), which qualitatively supports findings from previous studies^5,12,28^. However, advancement of leaf unfolding was particularly pronounced at higher elevations (e.g., since ca. 1980 for larch, horse chestnut, hazel and beech ≥ 800 m). Significant effects were found mainly for negative trends (Fig. 4). A shift to a larger percentage of stations with negative trends occurred from ca. 1970 to 1990 for species with longer series, as also found for Norway spruce in Germany^29^. Since the mid-1990s, a majority of the stations showed negative trends for all species, ranging from 64.5% for horse chestnut to 87.8% for beech in 2014 (Fig. 4). The observed shifts of the timing of leaf unfolding in the period 1970 to 1990 likely reflect trends in spring temperatures, which have decreased from 1950 to ca. 1975 (−0.050 ± 0.016 K/year in 1950; mean ± standard deviation of smoothed slope) and increased from ca. 1983 to 2011 (0.108 ± 0.010 K/year in 2011) across all stations (Supplementary Fig. S5). Thus, contrasting periods of climate cooling and climate warming have manifested as a temporal sign-switching^6^ in phenological trends. These observed shifts of leaf unfolding and spring temperatures are congruent with the phenomenon of global dimming and brightening, i.e. the large-scale decrease in surface solar radiation and the increase in aerosols between the 1950s and 1980s and the subsequent increase in solar radiation^30^.

The date safety margins of frost risk shifted from predominantly positive trends (i.e. increasing safety margins and decreasing frost risk) in the late 1950s and 1960s to increasingly higher amounts of negative trends (i.e. decreasing safety margins and increasing frost risk) since the 2000s (Fig. 4). At stations <800 m, some species showed mainly negative trends after ca. 1980 to 1990 (larch, horse chestnut, hazel, beech, Norway spruce), i.e. the time between the last spring frost and leaf unfolding decreased at these stations. Since the 2000s, several species showed positive trends at a majority of stations ≥1100 m (horse chestnut, hazel, silver birch, rowan, sycamore, large-leaved linden; Fig. 4), i.e. the time from the last late frost to leaf unfolding increased at these stations.

The temperature safety margins of frost risk shifted towards predominantly positive trends after the 1980s (Fig. 4). Since the 2000s, positive trends were recorded particularly at stations <800 m (larch, horse chestnut, hazel, silver birch, rowan, beech, Norway spruce), i.e. T_min_ during leaf unfolding increased for these species.

The overall analysis of frost trends showed for the last mild to moderate spring frosts (T_min_ < −1 °C to <−6 °C) negative trends particularly at elevations ≥800 m (Supplementary Fig. S6), i.e. the dates of these frosts advanced. The mildest spring frosts (T_min_ < −1 °C to <−3 °C) showed a pronounced shift to earlier dates starting in ca. 1980, which likely reflects the transition from global dimming to global brightening^30^. The last severe spring frosts (T_min_ < −7 °C to <−10 °C) shifted to a slightly higher percentage of stations with positive trends (Supplementary Fig. S6). This complex pattern of shifts across frost intensities and elevations in combination with the changed timing in leaf unfolding eventually determines the observed differential changes of date safety margins and temperature safety margins (Fig. 4).

We conclude that across many European temperate tree and shrub species, relatively large safety margins are found regarding frost risk during leaf unfolding. The safety margins increase from early- to late-leafing species and generally decrease with increasing elevation, i.e. early-leafing species at high-elevation sites generally feature the lowest safety margins. Severe spring frost occurred in only few cases during or after leaf unfolding. While some of these findings may be affected by the uncertainty of the data (i.e. phenological observations, interpolated weather data, and estimates of frost resistance), we still believe that the resulting patterns are robust since we analyzed data across a large environmental gradient over a relatively long period. The timing of phenological processes such as leaf unfolding is critical for survival, thus trees and shrubs have adapted to these seasonal climates over evolutionary time scales^1,7^. Because late spring frosts are strong selective forces in temperate climates, the onset of leaf unfolding relies on multiple environmental cues including chilling and forcing temperatures as well as daylength.

Overall, the timing of leaf unfolding reflects a trade-off between minimizing frost risk and maximizing the length of the growing period. While recent warming has induced phenological shifts of trees and shrubs to earlier leaf unfolding, the time between the last critical spring frost and leaf unfolding has shifted from positive trends to increasingly higher amounts of negative trends. Thus, under future global warming frost risk is likely to increase, particularly if more frequently occurring warm springs combine with severe frost events^11,14^ (Fig. 1). By applying a dynamic modeling framework, we revealed that many observed trends vary over time, in contrast to previous studies that relied on time-invariant trends, which have likely misestimated the actual trends underlying phenological observations, temperatures, or frost risk (cf. Fig. 2 and Supplementary Fig. S7). Such dynamic changes in trends (i.e. temporal sign-switching) are considered a strong fingerprint of a biotic response to climate cooling and warming^6^. Thus, climatic change results in time-varying shifts in leaf unfolding, spring temperatures and occurrence of frost events, which entail species-specific changes in frost risk over time as well as across elevation and thus temperature gradients. This improved understanding of the phenological dynamics in response to climate change and the timing of frost events is required to increase the accuracy of projected tree species distributions, biomass production in forest ecosystems, vegetation feedbacks to the climate system, and biogeochemical cycling^7,9,31^.

## Methods

### Phenological observations

We used dates of observed leaf unfolding from 13 tree and shrub species that are common and widespread in Europe (deciduous species: European beech - *Fagus sylvatica*, black locust - *Robinia pseudoacacia*, European aspen - *Populus tremula*, horse chestnut - *Aesculus hippocastanum*, large-leaved linden - *Tilia platyphyllos*, rowan - *Sorbus aucuparia*, silver birch - *Betula pendula*, small-leaved lime - *Tilia cordata*, sweet chestnut - *Castanea sativa*, sycamore - *Acer pseudoplatanus*; conifer species: European larch - *Larix decidua*, Norway spruce - *Picea abies*; shrub species: common hazel - *Corylus avellana*). The phenological observations are available from the phenology network of MeteoSwiss (the Swiss Federal Office of Meteorology and Climatology) and have been recorded by volunteers every year since 1951^28,32^. The dates of leaf unfolding have been recorded based on 1 to 3 visits per week when an estimated 50% of the leaves of one or several trees or shrubs were unfolded (i.e. leaf surface and leaf base visible in deciduous species, young needle bundles start to open and spread in conifer species)^33^. Missing data and incomplete series are common, and series from 7 of the 13 species started in 1996 only. We thoroughly checked the leaf unfolding data for plausibility and consistency, and classified 0.023% of the observations as outliers, which we thereafter treated as missing data. Finally, almost 48’000 observations were available from 1951 to 2014 (Supplementary Fig. S8) across 264 phenological stations in Switzerland (Supplementary Table S1 and Supplementary Fig. S9). The stations are distributed over ca. 41’000 km^2^ and extend over an elevational range from 200 to 1900 m a.s.l., thus covering sub-mediterranean to subalpine conditions and representing different climate regimes from oceanic climate (Cfb according to the Köppen-Geiger climate classification), warm humid continental climate (Dfb), subarctic climate (Dfc) to Tundra climate (ET). We converted the dates of leaf unfolding to the variable “doy” (day of year) with values ranging from 1 (1 January) to 365 (366 in leap years; 31 December).

### Weather data

We used spatially interpolated daily minimum air temperature (T_min_) and daily average air temperature (T_ave_) from 1950 to 2011 (no data were available after 2011) at each of the 264 phenological stations (Supplementary Fig. S9). These daily gridded weather data with a spatial resolution of 100 m were derived from measured data of MeteoSwiss climate stations and a digital elevation model using the DAYMET interpolation algorithm^34^. To obtain robust estimates, T_min_ and T_ave_ of the nearest grid cell and the eight neighboring cells were averaged for each station. These interpolated weather data were provided by the Landscape Dynamics group at the Swiss Federal Institute for Forest, Snow and Landscape Research WSL (Birmensdorf, Switzerland). We validated the interpolated T_min_ values for each phenological station with observed T_min_ values from the closest MeteoSwiss climate station. The median Pearson correlation based on daily values was r = 0.985, and the lowest correlation was r = 0.852.

### Last spring frosts and frost risk

The spatially explicit, interpolated estimates of T_min_ were used to extract the date of the last frost in spring for each year and each station. We defined frost events based on (1) fixed temperature thresholds to assess overall frost trends; and (2) species-specific, critical temperature thresholds (i.e. frost resistance) to assess frost risk. To extract the date of the last spring frost, we considered frost events in the period from doy 1–212 of the current year (i.e. 1 January to 30/31 July, depending on leap years) and doy 213–365/366 of the previous year (i.e. 31 July/1 August to 31 December, depending on leap years). If the last frost occurred in the previous year, we extended the series as doy = 0 (31 December), doy = −1 (30 December) etc. We treated years to have missing data if no T_min_ was observed below a given temperature threshold.

To select frost events based on fixed temperature thresholds, we used ten negative temperature thresholds (T_min_ < −1 °C, …, <−10 °C). The last spring frosts were used to assess overall frost trends across Switzerland, i.e. we tested whether the date of the last spring frost changed systematically through time. For all 264 stations and each year from 1950 to 2011, we recorded separately for each of the 10 temperature thresholds when the last frost occurred with T_min_ below the corresponding threshold.

To select frost events based on species-specific critical temperature thresholds, we used estimates of frost resistance for all 13 tree and shrub species (see below). Frost resistance, dates of leaf unfolding and T_min_ were then used to assess frost risk represented by (1) the date safety margin, and (2) the temperature safety margin, as explained below. Frost resistance is often expressed as a critical temperature, i.e. the temperature when 50% of the leaves get killed (lethal temperature, LT_50_). Frost resistance of buds and leaves changes through the season^3,35^ and is typically highest in winter (i.e., low LT_50_) and lowest during leaf unfolding (i.e., high LT_50_)^3^. Frost resistance is commonly determined in the field or in the lab using an electrolyte leakage test or a visual damage assessment. We conducted an extensive literature search and compiled published estimates of LT_50_ during leaf unfolding for as many species as possible. Although estimates of LT_50_ vary to some extent among populations^36,37^, trees^3,38^, and measurement methods^39,40^, we posit that the published estimates of LT_50_ are useful to characterize frost resistance. We found for 6 of 13 species estimates of LT_50_ (Supplementary Fig. S10). Since for 5 of these 6 species two or three estimates of LT_50_ were available each and since there were no published LT_50_ values for the remaining 7 species, we used a non-linear regression model to estimate LT_50_ for all 13 species. We assumed that early-leafing species have a higher frost resistance during leaf unfolding since they are more likely exposed to severe frosts than late-leafing species^3^. Previous studies have confirmed this assumption using linear relationships between dates of leaf unfolding and estimates of LT_50_^3,38^. To ensure comparability, we extracted for each species the median date (doy) of leaf unfolding over those 34 stations where all species were observed, and over the common period covered by all species (1996 to 2011 except for European aspen, where 1980 to 1995 had to be used; Fig. 3a). The order of the median dates based on all 264 stations was reasonably stable also across elevation bands (Supplementary Fig. S1). To derive meaningful estimates of LT_50_, we used two asymptotes to restrict the estimated values to below −1 °C, which corresponds approximately to the highest observed LT_50_ of woody plants in temperate climates^41^, and above −7.5 °C, which is slightly below the lowest LT_50_ value found for the species studied here. We used a four-parameter logistic model^42^ with two fixed asymptotes and two free parameters to estimate LT_50_ from the median dates of leaf unfolding (Supplementary Fig. S10):

1 $$L{T}_{50}={\varphi }_{1}+\frac{{\varphi }_{2}-{\varphi }_{1}}{1+\exp [({\varphi }_{3}-x)/{\varphi }_{4}]}$$

where ϕ_1_ = −1 and ϕ_2_ = −7.5 are fixed parameters, ϕ_3_ and ϕ_4_ are free parameters (estimate ± standard error: ϕ_3_ = 127.158 ± 3.535, ϕ_4_ = −9.249 ± 3.007), and x is the predictor variable (median date of leaf unfolding). The choice of ϕ_1_ and ϕ_2_ did not strongly affect estimated LT_50_ values (e.g., setting ϕ_1_ = 0 and ϕ_2_ = −15 changed the estimated LT_50_ values by only −0.25 K to 0.31 K). We fitted the non-linear model using the function “nls” in the package “nlme” (version 3.1–131)^42^ of the statistical software R (version 3.3.1)^43^.

Species-specific frost resistance values (i.e., estimated LT_50_; Supplementary Fig. S10) were used to assess frost risk, which was represented by the date safety margin and the temperature safety margin. We defined the date safety margin^22,23^ (DSM) as:

2 $${\rm{DSM}}={{\rm{doy}}}_{{\rm{leaf}}{\rm{unfolding}}}-{{\rm{doy}}}_{{\rm{last}}{\rm{frost}}}$$

where doy_leaf unfolding_ is the date of leaf unfolding and doy_last frost_ is the date of the last spring frost with T_min_ < LT_50_ (for an example see Supplementary Fig. S11). A positive date safety margin indicates that leaf unfolding occurred after the last spring frost, whereas a negative date safety margin indicates that the last spring frost occurred after leaf unfolding, which results in significant frost damage.

We defined the temperature safety margin^3,22^ (TSM) as

3 $${\rm{TSM}}={{\rm{T}}}_{{\rm{low}}}-{{\rm{LT}}}_{50}$$

where T_low_ is the lowest T_min_ within 5 days before and after doy_leaf unfolding_ (for an example see Supplementary Fig. S11). A positive temperature safety margin indicates that the minimum temperatures during leaf unfolding are above the species-specific, critical temperature (LT_50_), whereas a negative temperature safety margin indicates that the lowest minimum temperatures during leaf unfolding are below LT_50_, which results in significant frost damage.

### Data analyses

Many studies dealing with the detection of trends (i.e. changes in a system over time^27^) rely on linear models (LMs), which are considered a parametric trend detection method:

4 $${y}_{t}={\beta }_{0}+{\beta }_{1}t+{\varepsilon }_{t},{\varepsilon }_{t}\, \sim N(0,{\sigma }^{2})$$

where y_t_ is the response variable at time t = {1, …, n}, β_0_ the intercept, and β_1_ the slope of the trend (i.e. change in y_t_ per unit change in t). The residual errors ε_t_ are assumed to be normally distributed random variables with a mean of zero and variance σ^2^. Trend analyses from environmental sciences using LMs are common and include weather data (e.g., changes in temperature over time^44^ or changes in the occurrence of the last spring frost^26,45^) and phenological observations (e.g., changes in dates of leaf unfolding^5,24^). However, trends in environmental systems are likely to change over time, which LMs cannot account for because the slopes of the trends are time-invariant, i.e. constant over time. To depict time-varying trends in phenological observations, some studies have used alternative methods such as polynomial linear models, spline smoothing or Wavelet analysis (for an overview see ref.^46^). However, for some of these methods, the slope of the trend cannot be readily extracted (but cf. the Bayesian change point model, which allows to estimate the derivative^47^) or lacks a measure of uncertainty.

We use dynamic linear models (DLMs), a stochastic trend detection method within the broad class of state-space models^27,48^. State-space models address a wide range of problems in time series analysis. In particular, they allow for (1) time-varying trends and explicit modeling of the slope of the trend; (2) non-stationary time series (i.e. both mean and variance can change over time); (3) missing data; and (4) separation of the process error (e.g., due to changing environmental conditions) from the observation error (e.g., due to differences in training of or methods adopted by the phenological observers). The main goal of state-space models is to obtain estimates of the unknown state of a system given a series of observations.

In the state-space modeling context, the observations y_t_ with t = {1, …, n} are described by the observation equation^48^:

5 $${y}_{t}={\mu }_{t}+{\varepsilon }_{t},\,{\varepsilon }_{t} \sim N(0,{\sigma }_{\varepsilon }^{2})$$

where y_t_ depends on the level of the state (μ_t_) at time t, and ε_t_ is the observation error with a mean of zero and variance ${\sigma }_{\varepsilon }^{2}$.

We used two state equations to describe the process^48,49^, i.e. the unobserved state $${\alpha }_{t}\,=\,(\begin{array}{c}{\mu }_{t}\\ {\nu }_{t}\end{array})$$:

6 $${\mu }_{t+1}={\mu }_{t}+{\nu }_{t}+{\xi }_{t},\,{\xi }_{t} \sim N(0,{\sigma }_{\xi }^{2})$$

7 $${\nu }_{t+1}=\,{\nu }_{t}+\,{\zeta }_{t},\,{\zeta }_{t} \sim N(0,{\sigma }_{\zeta }^{2})$$

where μ_t+1_ describes the level of the state at time t + 1, which depends on the level of the state at time t (μ_t_) and the slope of the state at time t (ν_t_). ν_t+1_ is the slope of the state at time t + 1. ${\xi }_{t}$ and ${\zeta }_{t}$ are state errors with a mean of zero and variances ${\sigma }_{\xi }^{2}$ and ${\sigma }_{\zeta }^{2}$, respectively.

The prior distribution for the initial state α_1_ was defined as:

8 $${\alpha }_{1} \sim N({a}_{1},{P}_{1})$$

We initialized the state α_1_ using relatively wide prior distributions: for the mean a_1_ we used y_1_ for the level and $\frac{1}{n-1}\sum _{i=1}^{n-1}({y}_{i+1}-{y}_{i})$ for the slope; for the variance P_1_ we used a value of 100 for the level and a value of 2 for the slope. This system of equations (equations 5–8) is also known as the local linear trend model with stochastic level and slope components^27,48^. The slope ν_t_ in the DLM (equation 7), which is allowed to change over time, corresponds to the slope β_1_ in the LM (equation 4), which is fixed over time.

We used maximum likelihood to estimate the parameters of the DLMs. The likelihood function was evaluated using the Kalman filtering algorithm^50^, which was applied to the observations Y_t_ = {y_1_, …, y_t_} to estimate the filtered states a_t+1_ = E(α_t+1_|Y_t_) and the filtered state variances P_t+1_ = Var(α_t+1_|Y_t_), i.e. only past and current observations were used. We then applied a Kalman smoothing algorithm to the filtered states and variances to estimate the smoothed states ${\hat{\alpha }}_{t}=E({\alpha }_{t}\,\,|\,\,{Y}_{n})$ and the smoothed state variances V_t_ = Var(α_t_|Y_n_), i.e. all observations Y_n_ = {y_1_, …, y_n_} with n ≥ t were used. In this study, we report on estimates of the smoothed states and variances for both levels and slopes (see example for leaf unfolding in Fig. 2). For further details about state-space models and the filtering and smoothing algorithms we refer to the literature^48,49^. We fitted the DLMs using the package “dlm” (version 1.1–4)^51^ of the statistical software R.

For the trend analyses, we fitted DLMs to the (1) dates of leaf unfolding; (2) date safety margins (DSM; equation 2); (3) temperature safety margins (TSM; equation 3); (4) mean spring temperatures (mean of daily T_ave_ from doy 75–125); and (5) last spring frost events (based on fixed temperature thresholds T_min_ < −1 °C, …, < −10 °C). We fitted univariate DLMs, i.e. separate models for the time series of each station. Only series with at least 5 observations were considered. Based on the smoothed slopes of the DLMs, we calculated the percentage of stations with (significantly) positive or negative trends over time. We assessed the significance of the trends based on 95% confidence intervals. To compare trends across elevations, we assigned the stations to four elevation bands: <500 m, 500–799 m, 800–1099 m, and ≥1100 m. Since the beginning and end of the phenological observations vary across stations, which may lead to artefacts (e.g., short series are more likely to exhibit a positive or negative trend during specific periods), the plots for leaf unfolding, date safety margin and temperature safety margin were constrained to those stations that had complete series of smoothed states from 1956 (1996 for species with later beginning of observations) to 2011 (2014 for leaf unfolding; 1995 for European aspen). Thus, any change in the percentage of stations with positive or negative trends is due to a change in the sign (i.e. temporal sign-switching) of the trend at individual stations. However, to warrant transparency, plots based on all series are shown in the Supplementary information as well. For comparability, the trend analyses for the dates of leaf unfolding and the safety margins based on DLMs (equations 5–8) were complemented with trend analyses based on LMs (equation 4). We created the figures using the package “ggplot2” (version 2.2.1) of the statistical software R.

### Data availability

The daily weather data from the MeteoSwiss climate stations as input for the DAYMET software (https://daymet.ornl.gov) and the phenological observations are available from IDAWEB (https://gate.meteoswiss.ch/idaweb).

## Electronic supplementary material

Supplementary information

Supplementary Table S1
