# Supplementary material for: Climate-induced shifts in leaf unfolding and frost risk of European trees and shrubs
Source: Sci Rep. 2018 Jun 29;8:9865. doi: 10.1038/s41598-018-27893-1 (PMC6026133; doi:10.1038/s41598-018-27893-1)
Supplement: Supplementary file 1 — Supplementary information [file 41598_2018_27893_MOESM1_ESM.pdf]

Supplementary information:

Climate-induced shifts in leaf unfolding and frost risk of European trees and shrubs

Christof Bigler[1]* and Harald Bugmann[1]

[1]Forest Ecology, Institute of Terrestrial Ecosystems, Department of Environmental Systems Science, ETH Zurich, Zurich, Switzerland

*e-mail: christof.bigler@env.ethz.ch

Supplementary Table S1 | Description of phenological stations and leaf unfolding data. For each station, the station code and station name as well as WGS84 coordinates (latitude, longitude) and elevation are shown. For each species, the number of recorded dates of leaf unfolding from 1951 to 2014 are indicated. See Supplementary Fig. S9 for a map of the stations.

(data can be found in the separate file “Supplementary_Table_S1.xls”)

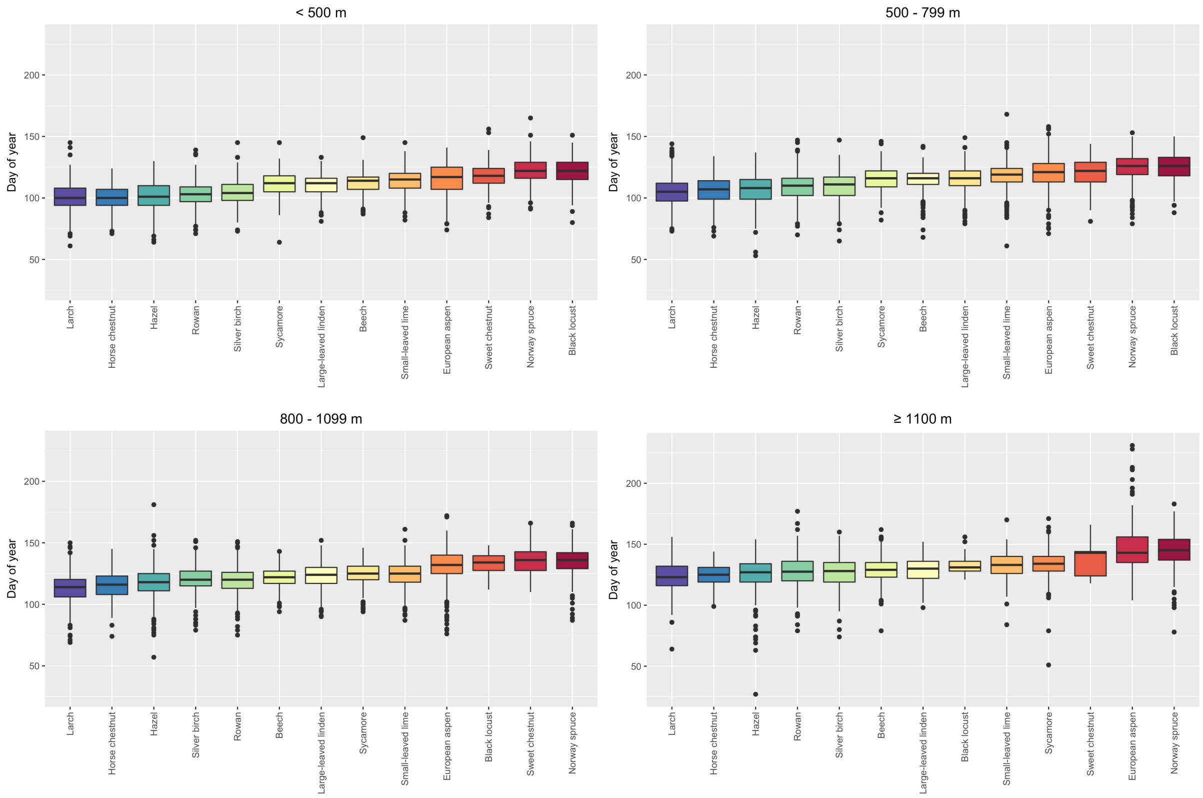

Supplementary Figure S1 | Distribution of leaf unfolding dates in different elevation bands. Box plots of observations from all stations for the common period (1996 to 2011; 1980 to 1995 for European aspen). Total number of observations per elevation band: < 500 m, n=6’399; 500-799 m, n=7’628; 800-1099 m, n=3’897; ≥ 1100 m, n=3’253. Within each elevation band, the species are ordered from early- (blue) to late-leafing species (red) according to median dates of leaf unfolding.

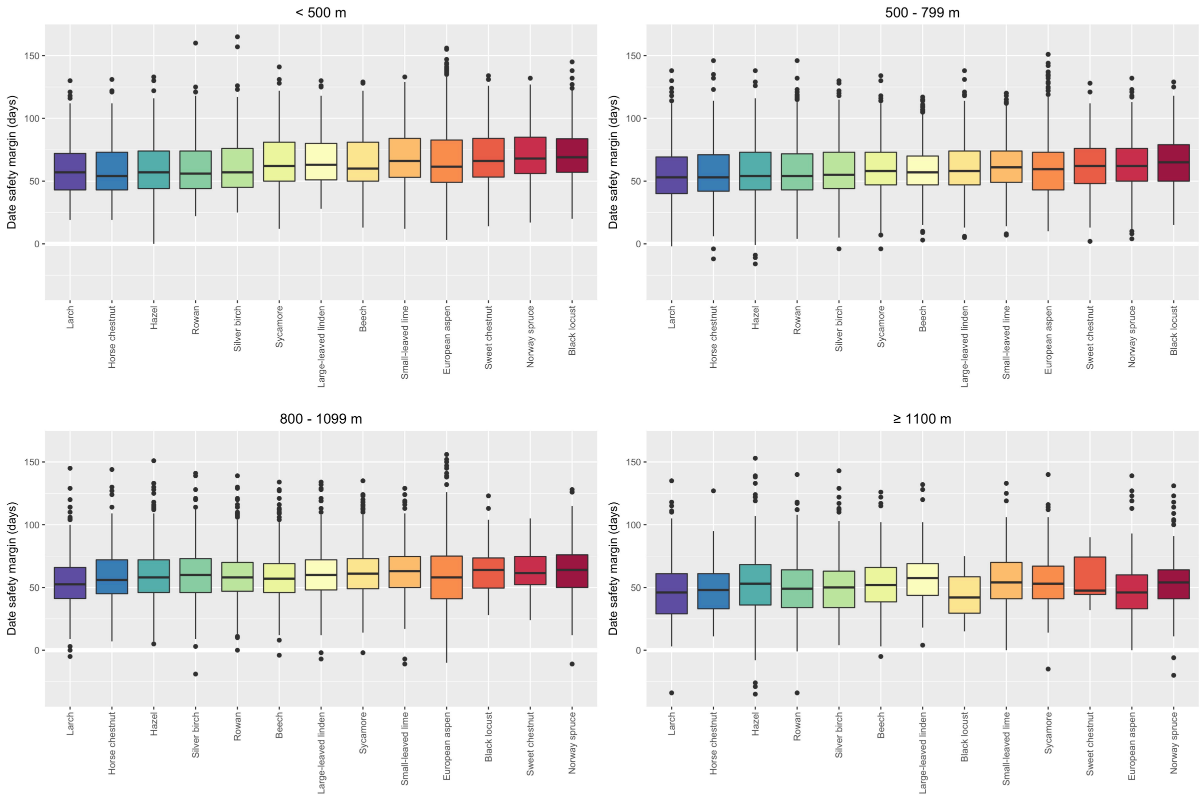

Supplementary Figure S2 | Distribution of date safety margins in different elevation bands. Box plots of observations from all stations for the common period (1996 to 2011; 1980 to 1995 for European aspen). Total number of observations per elevation band: < 500 m, n=5’911; 500-799 m, n=7’537; 800-1099 m, n=3’886; ≥ 1100 m, n=3’249. Within each elevation band, the species are ordered from early- (blue) to late-leafing species (red) according to median dates of leaf unfolding (see Supplementary Fig. S1).

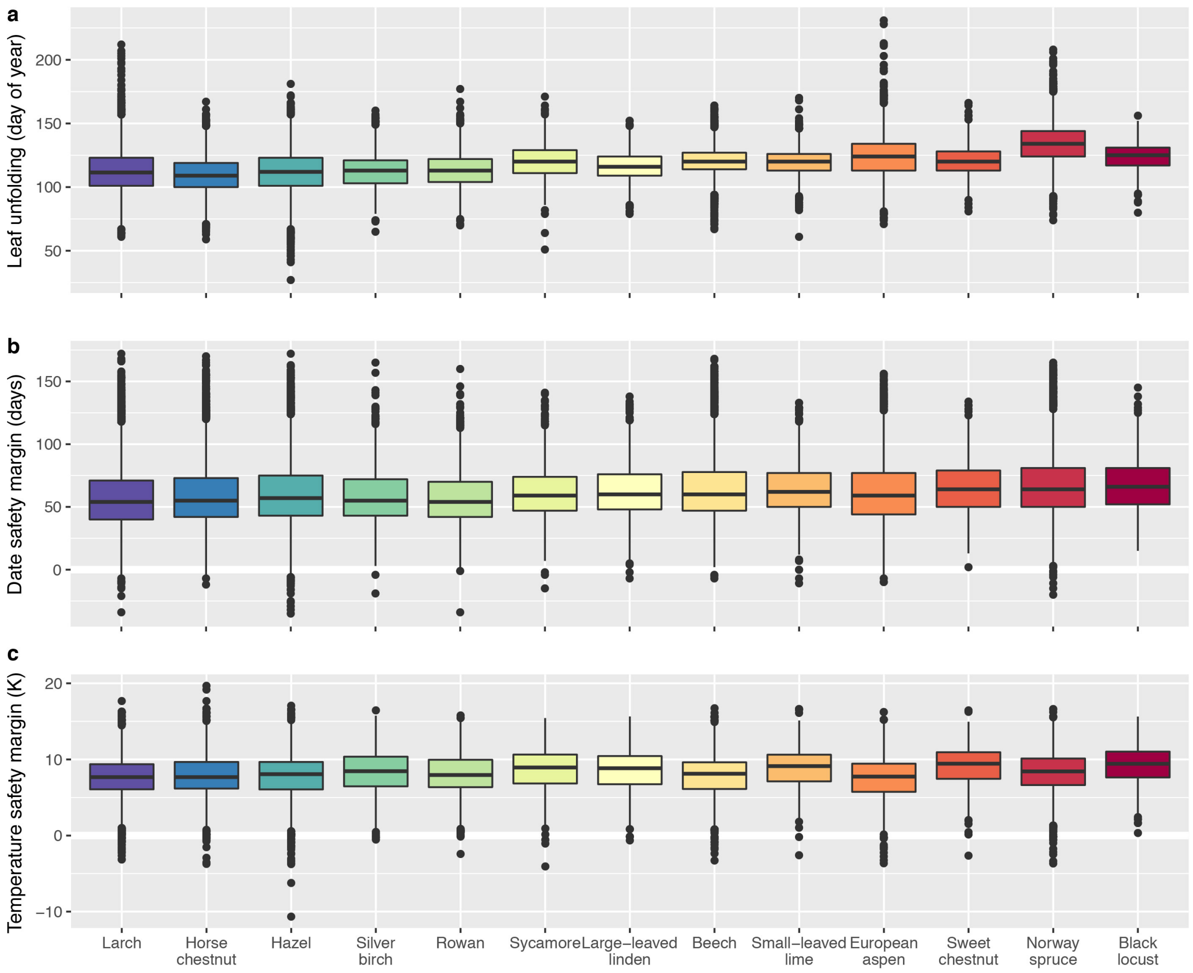

Supplementary Figure S3 | Distributions of leaf unfolding dates and frost risk (date safety margin and temperature safety margin). Box plots of: **a**, leaf unfolding dates, **b**, date safety margin (see equation 2), and **c**, temperature safety margin (see equation 3). Observations from 1951 to 2011 at all stations are shown (number of observations: leaf unfolding, n=44’242; date safety margin, n=42’889; temperature safety margin, n=44’242). The species are ordered from early- (blue) to late-leafing species (red) according to median dates of leaf unfolding (see Fig. 3a).

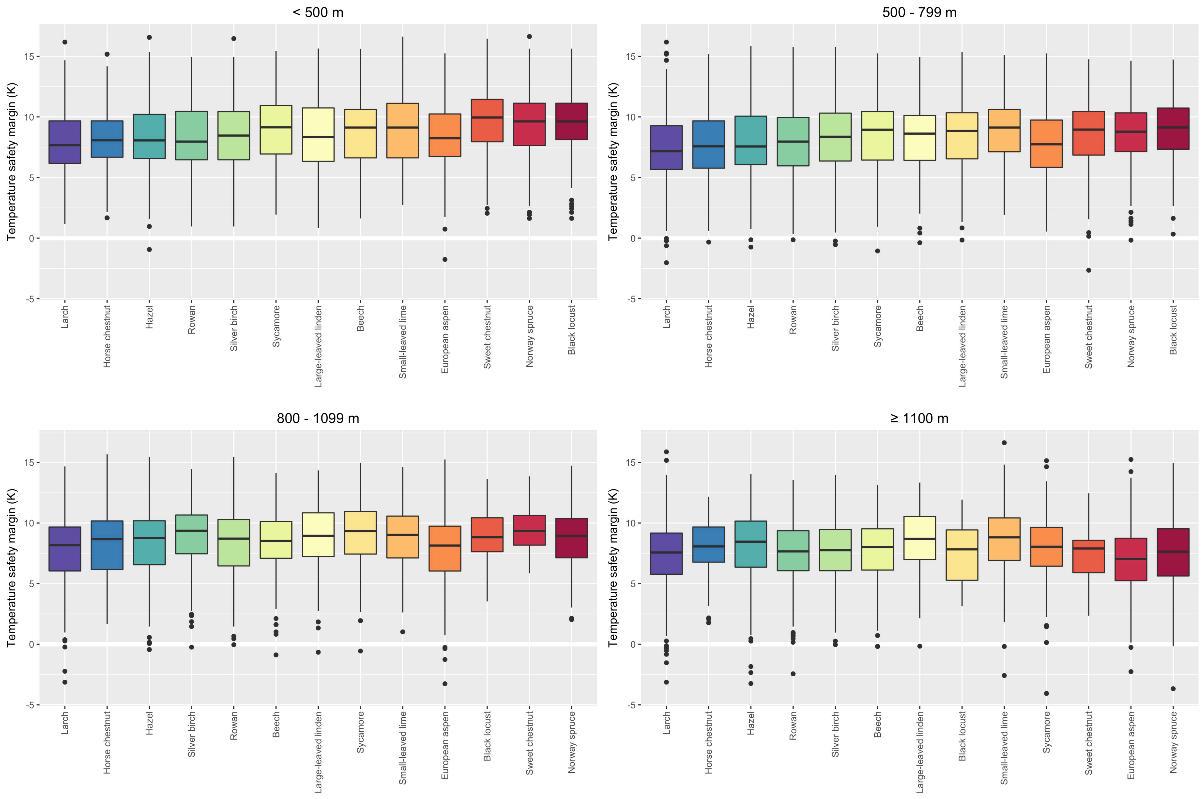

Supplementary Figure S4 | Distribution of temperature safety margins in different elevation bands. Box plots of observations from all stations for the common period (1996 to 2011; 1980 to 1995 for European aspen). Total number of observations per elevation band: < 500 m, n=6’399; 500-799 m, n=7’628; 800-1099 m, n=3’897; ≥ 1100 m, n=3’253. Within each elevation band, the species are ordered from early- (blue) to late-leafing species (red) according to median dates of leaf unfolding (see Supplementary Fig. S1).

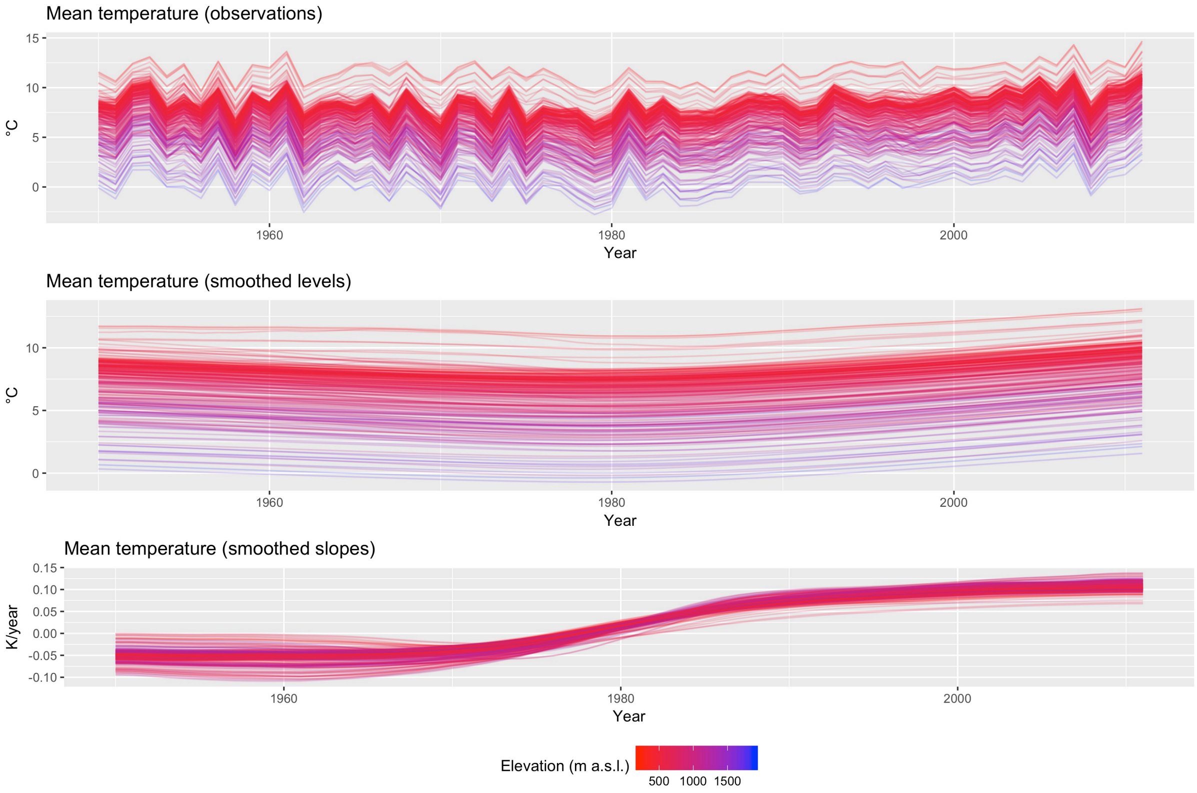

Supplementary Figure S5 | Observations, smoothed levels and smoothed slopes of mean spring temperature. Mean temperature was calculated as the mean of daily T_{ave} over doy 75 - 125. The smoothed levels and smoothed slopes are based on DLMs (see equations 5-8). The color gradient represents station elevation (see Supplementary Table S1).

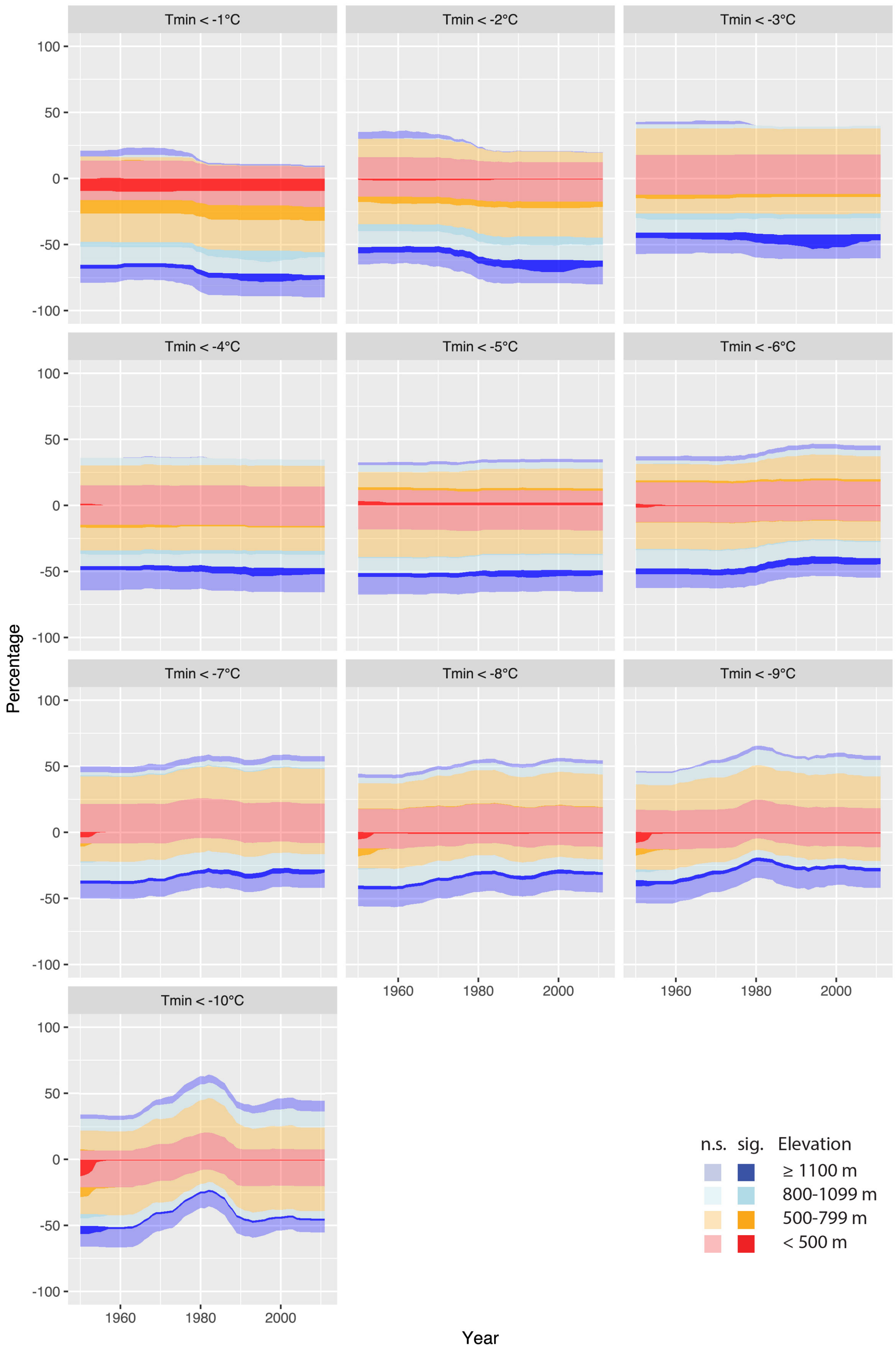
Tmin < -1°C
Tmin < -2°C
Tmin < -3°C
Tmin < -4°C
Tmin < -5°C
Tmin < -6°C
Tmin < -7°C
Tmin < -8°C
Tmin < -9°C
Tmin < -10°C
Percentage
100
50
0
-50
-100
1960
1980
2000
Year
n.s. sig. Elevation
≥ 1100 m
800-1099 m
500-799 m
< 500 m

Supplementary Figure S6 | Shifts in last late frost events. Percentages of stations assigned to four elevation bands with positive smoothed slopes (positive percentages on y-axis) and negative smoothed slopes (negative percentages on y-axis) based on DLMs (see equations 5-8). The DLMs were fitted to last spring frosts based on fixed temperature thresholds T_{min} < -1 °C, …, < -10 °C. For each temperature threshold, T_{min} from 1951 to 2011 at all 264 stations was used. Non-significant slopes (n.s.) are shown with semi-transparent colors, significant slopes (sig.) with opaque colors. For each year, the absolute values of positive and negative percentages across all elevation bands sum up to 100 %.

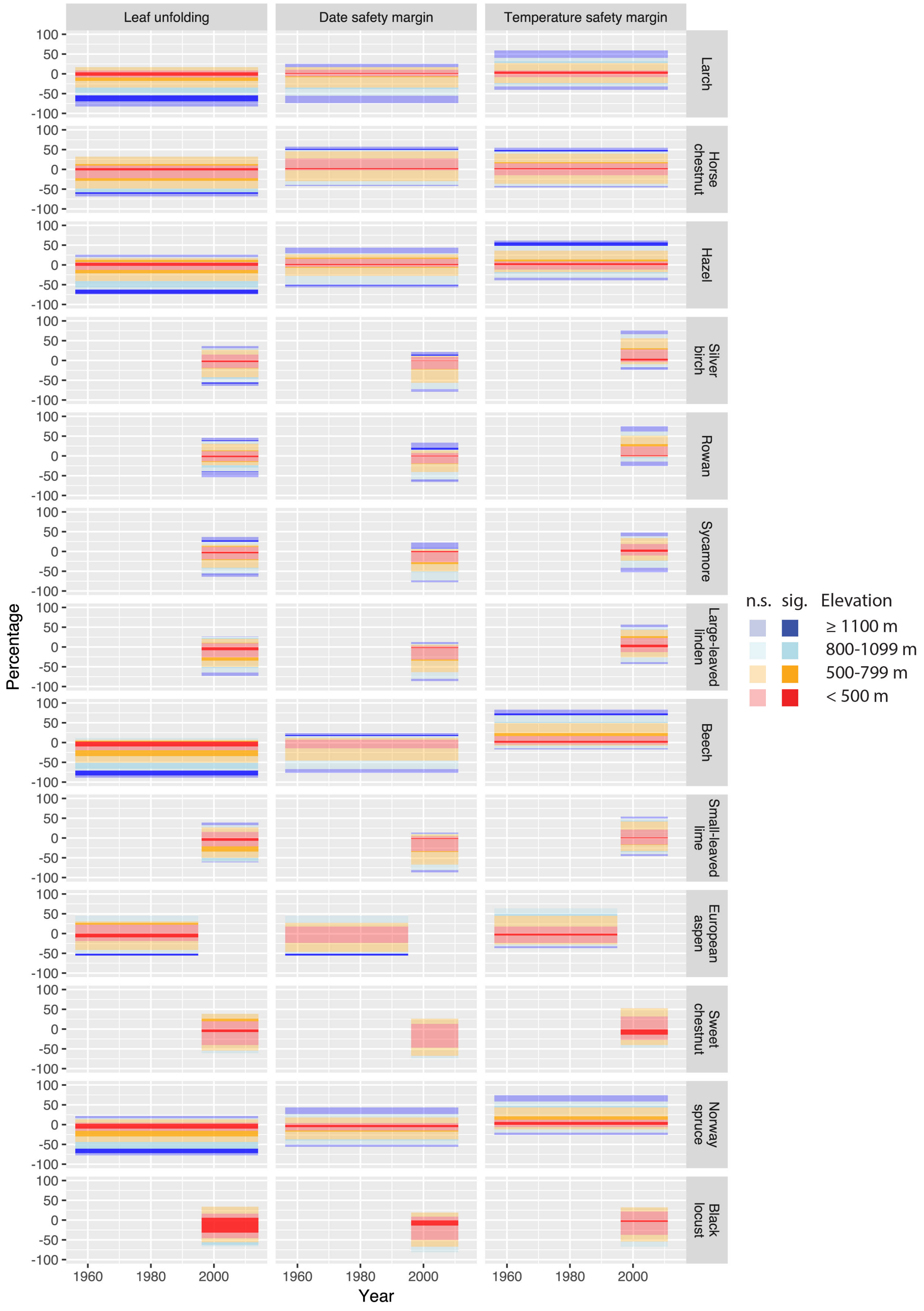
Leaf unfolding
Date safety margin
Temperature safety margin
Larch
Horse chestnut
Hazel
Silver birch
Rowan
Sycamore
Large-leaved linden
Beech
Small-leaved lime
European aspen
Sweet chestnut
Norway spruce
Black locust
Percentage
100
50
0
-50
-100
1960
1980
2000
Year
n.s.
sig.
Elevation
≥ 1100 m
800-1099 m
500-799 m
< 500 m

Supplementary Figure S7 | Shifts in leaf unfolding dates and frost risk (date safety margin and temperature safety margin) based on linear models (LMs). Percentages of stations assigned to four elevation bands with positive slopes (positive percentages on y-axis) and negative slopes (negative percentages on y-axis) based on LMs (see equation 4). The species are ordered according to median dates of leaf unfolding (see Fig. 3a). Only series from 1956 (1996 for species with later beginning of observations) to 2011 (2014 for leaf unfolding; 1995 for European aspen) without missing estimates of slopes are shown (number of series: larch, n=46-47; horse chestnut, n=31-33; hazel, n=39-44; silver birch, n=74-78; rowan, n=90-94; sycamore, n=79; large-leaved linden, n=70-73; beech, n=41-42; small-leaved lime, n=63-64; European aspen, n=22; sweet chestnut, n=15; Norway spruce, n=35-36; black locust, n=29-30). Non-significant slopes (n.s.) are shown with semi-transparent colors, significant slopes (sig.) with opaque colors. For each species and year, the absolute values of positive and negative percentages across all elevation bands sum up to 100 %.

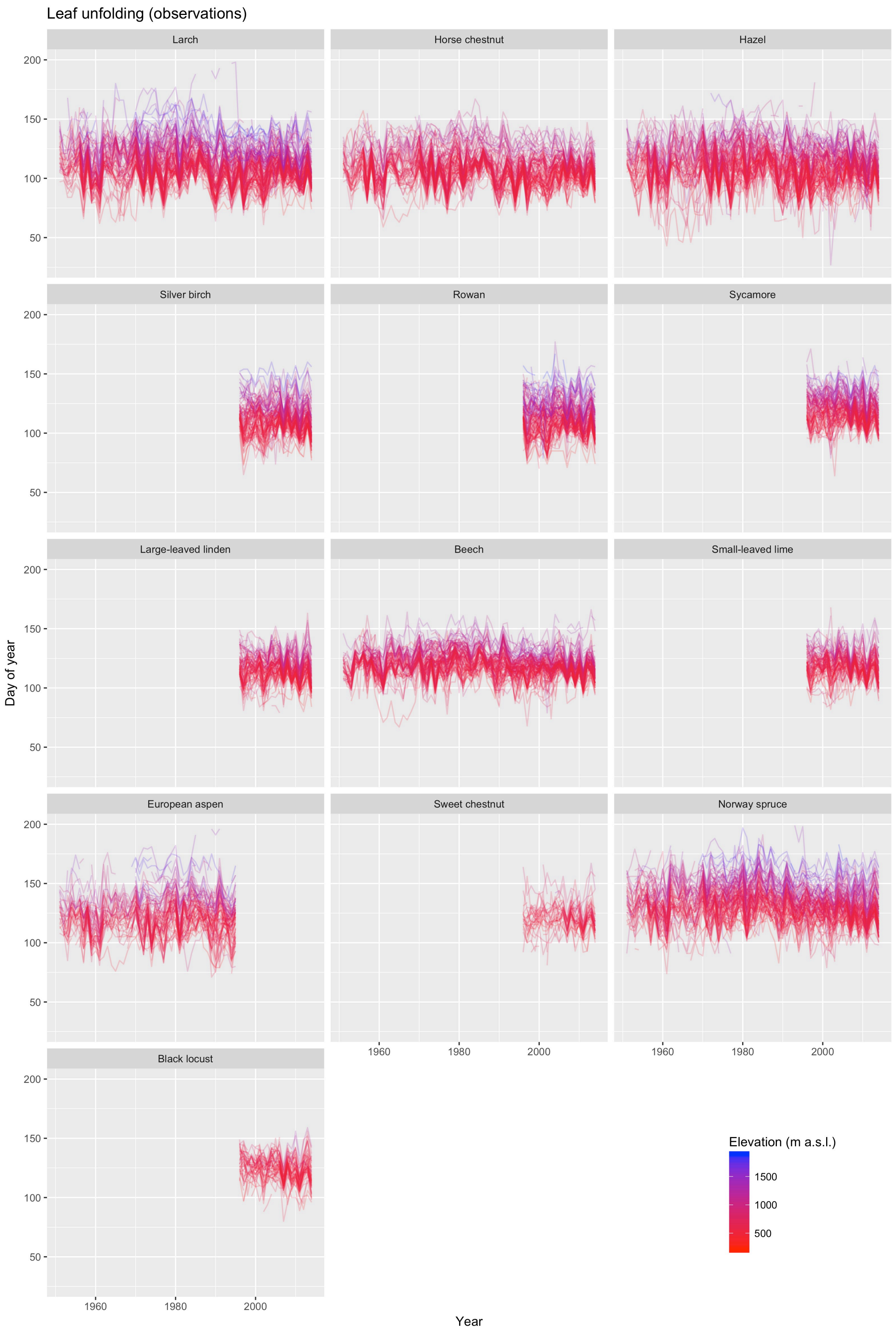
Leaf unfolding (observations)
Larch
Horse chestnut
Hazel
Silver birch
Rowan
Sycamore
Large-leaved linden
Beech
Small-leaved lime
European aspen
Sweet chestnut
Norway spruce
Black locust
Day of year
200
150
100
50
1960
1980
2000
Year
Elevation (m a.s.l.)
1500
1000
500

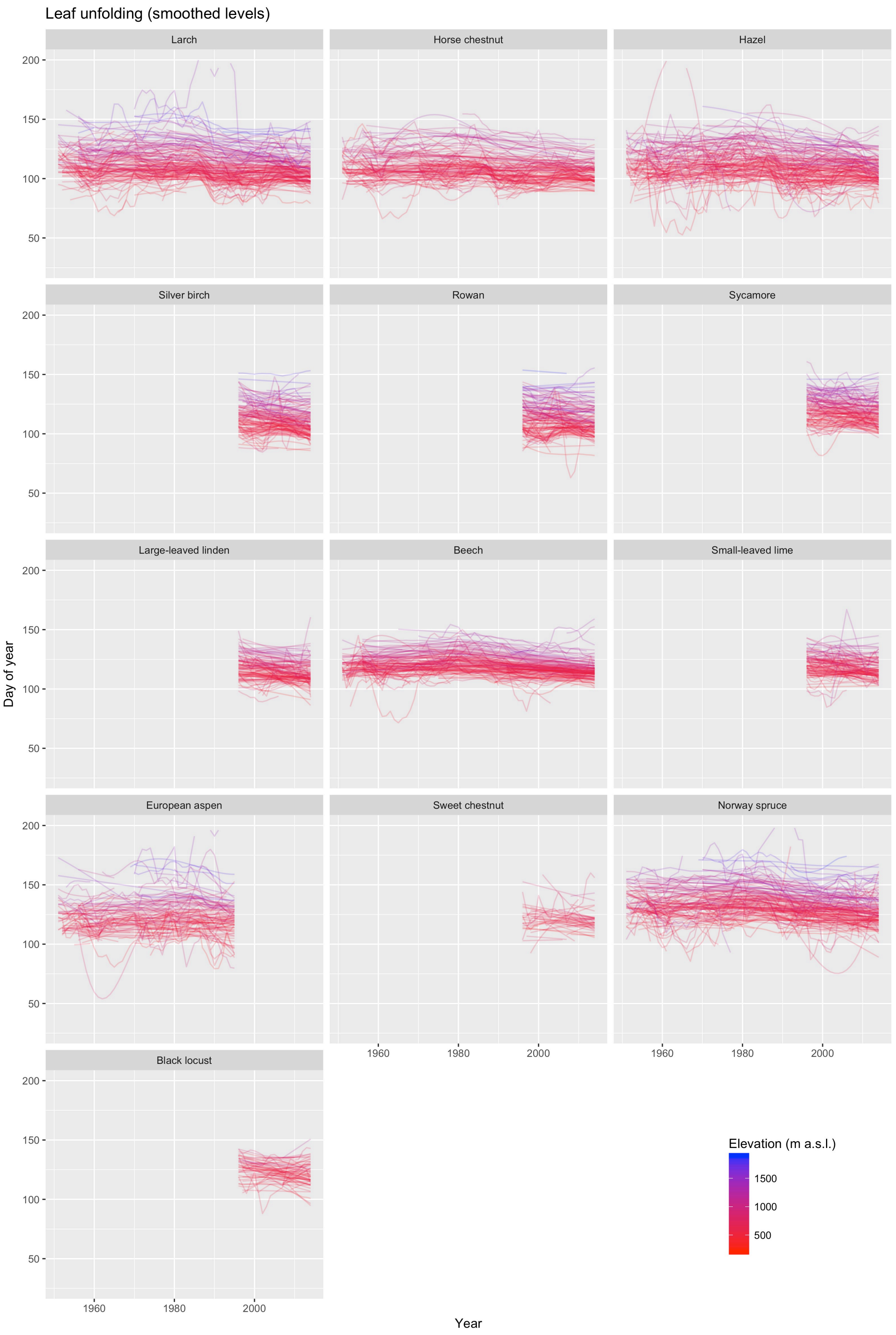
Leaf unfolding (smoothed levels)
Larch
Horse chestnut
Hazel
Silver birch
Rowan
Sycamore
Large-leaved linden
Beech
Small-leaved lime
European aspen
Sweet chestnut
Norway spruce
Black locust
Day of year
Year
200
150
100
50
1960
1980
2000
Elevation (m a.s.l.)
1500
1000
500

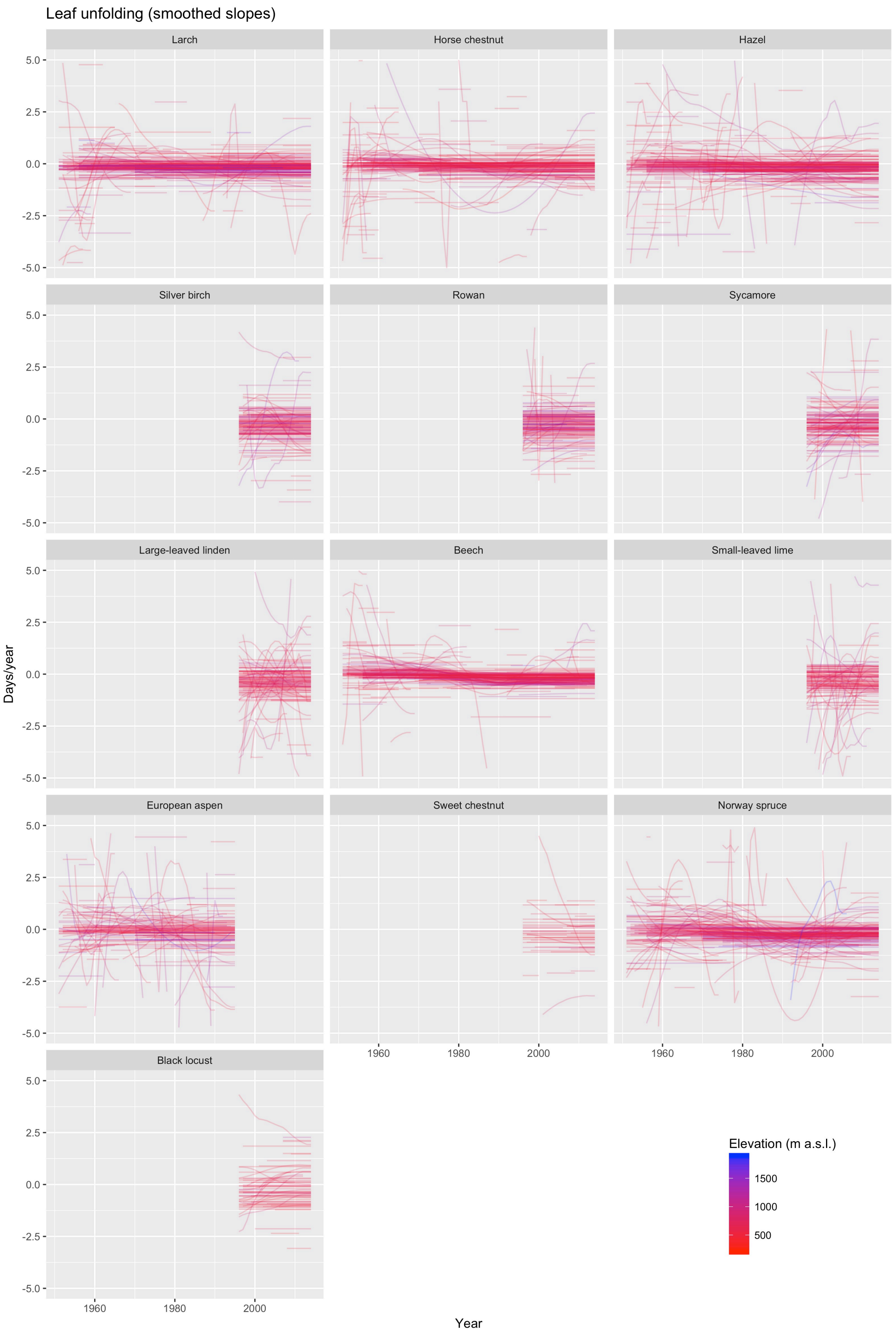
Leaf unfolding (smoothed slopes)
Larch
Horse chestnut
Hazel
Silver birch
Rowan
Sycamore
Large-leaved linden
Beech
Small-leaved lime
European aspen
Sweet chestnut
Norway spruce
Black locust
5.0
2.5
0.0
-2.5
-5.0
Days/year
1960
1980
2000
Year
Elevation (m a.s.l.)
1500
1000
500

Supplementary Figure S8 | Observations, smoothed levels and smoothed slopes of leaf unfolding. The smoothed levels and smoothed slopes are based on DLMs (see equations 5-8). The species are ordered according to median dates of leaf unfolding (see Fig. 3a). The color gradient represents station elevation (see Supplementary Table S1). For clarity, the y-axes were restricted to [25, 200] for the observations (< 0.03 % of data are omitted) and the smoothed levels (< 0.06 % omitted) and to [-5, 5] for the smoothed slopes (< 0.55 % omitted).

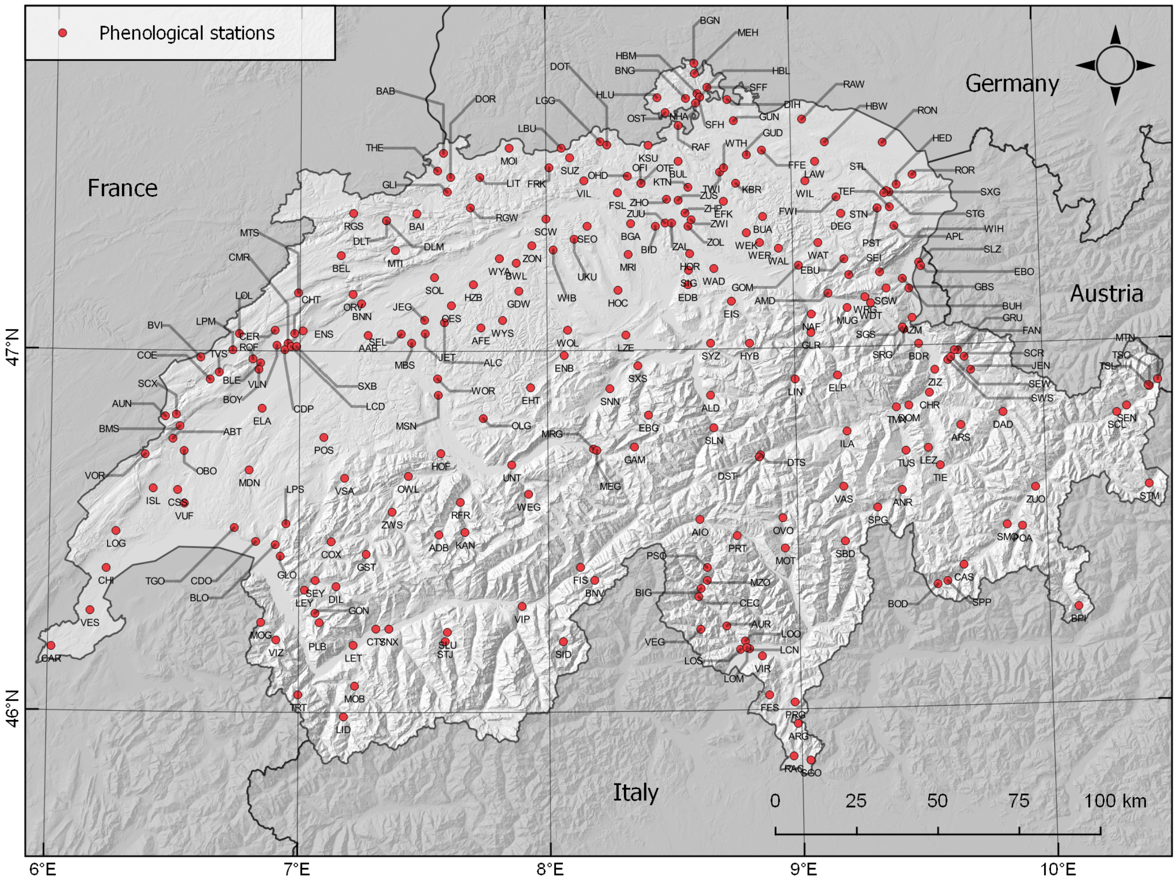

Supplementary Figure S9 | Location of phenological stations. For station codes and further information see Supplementary Table S1.

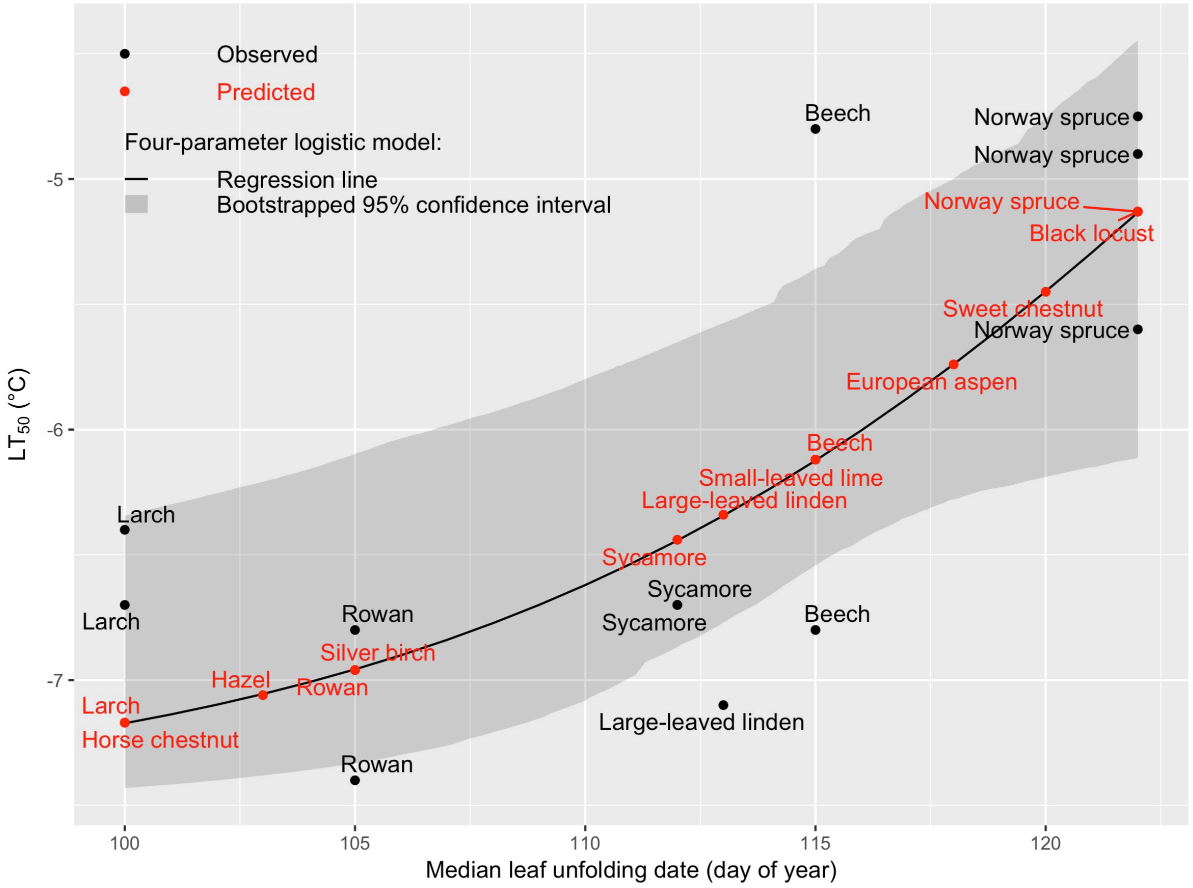

Supplementary Figure S10 | Non-linear relationship between median dates of leaf unfolding and frost resistance (LT_{50}). Based on published estimates of LT_{50} (black dots) and the median dates of leaf unfolding (doy, day of year) at the 34 common stations (see Fig. 3a), a four-parameter logistic model was fitted (black line; see methods). The approximate 95 % confidence interval (grey) was based on bootstrap resampling with 9999 samples. Estimates of LT_{50} during leaf unfolding were available for beech[1-3], large-leaved linden[3], larch[4,5], Norway spruce[4,6], rowan[1,2,4], and sycamore[1-3]. The predicted values of LT_{50} (red dots) were estimated for all species (larch and horse chestnut: -7.17 °C; hazel: -7.06 °C; silver birch and rowan: -6.96 °C; sycamore: -6.44 °C; large-leaved linden: -6.34 °C; beech and small-leaved lime: -6.12 °C; European aspen: -5.74 °C; sweet chestnut: -5.45 °C; Norway spruce and black locust: -5.13 °C).

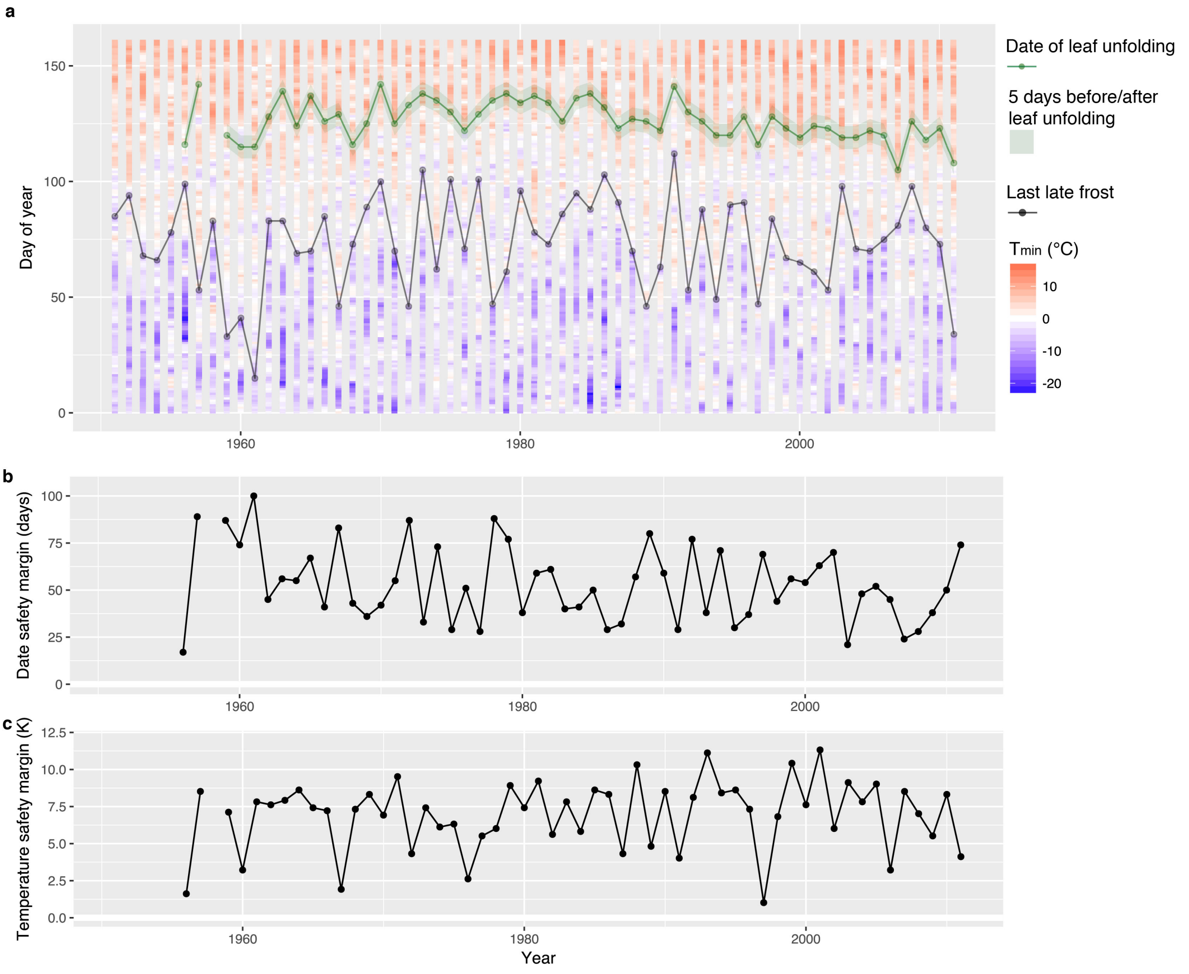

Supplementary Figure S11 | Leaf unfolding and calculation of safety margins. Data for beech at the station La Valsainte (see Supplementary Table S1 and Supplementary Fig. S9) are used to illustrate the pattern: **a**, observed dates of leaf unfolding, period of 5 days before and after leaf unfolding, last late frost events based on LT_{50} (-6.12 °C) for beech, daily minimum temperature T_{min}; **b**, date safety margin (equation 2); **c**, temperature safety margin (equation 3).

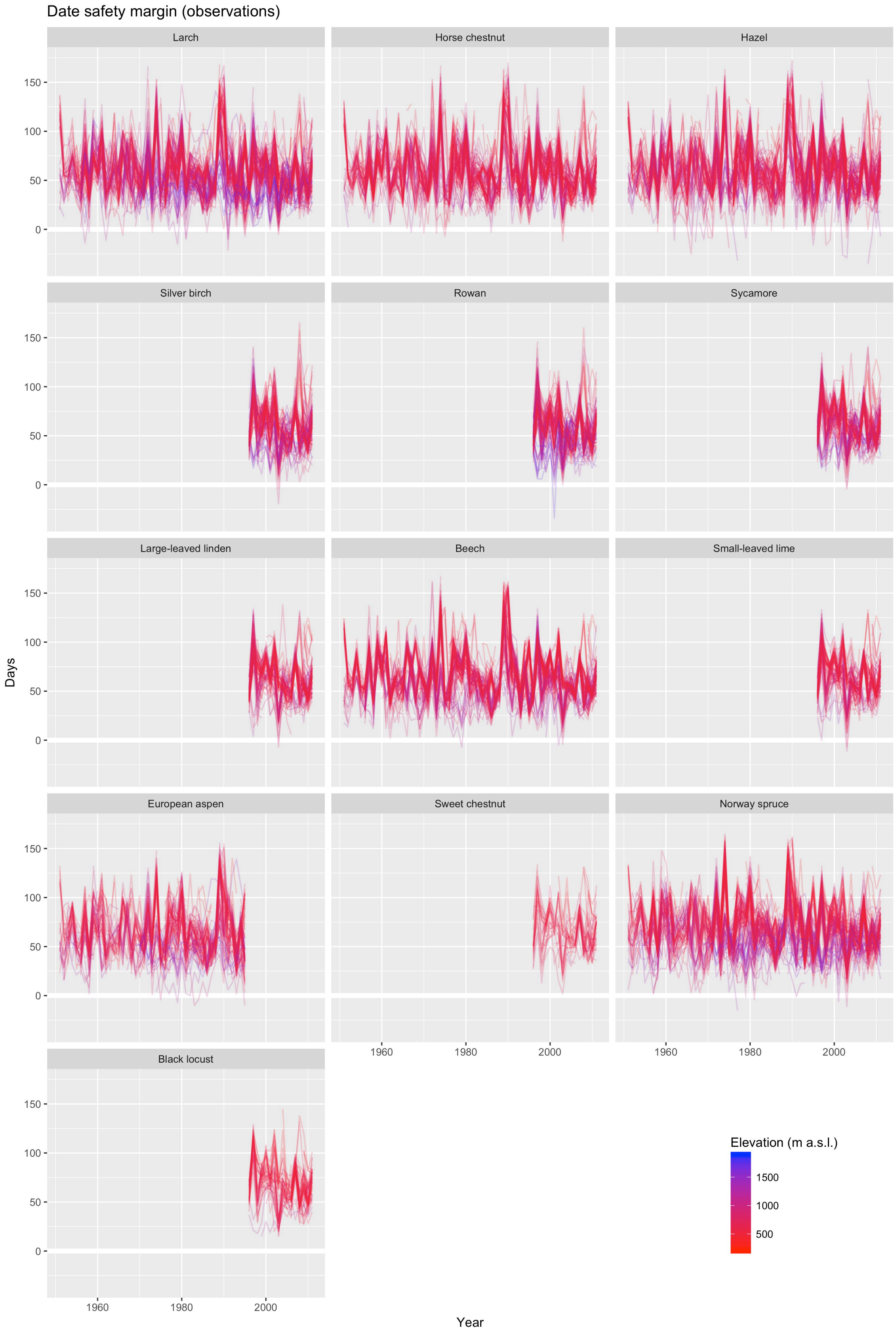
Date safety margin (observations)
Larch
Horse chestnut
Hazel
Silver birch
Rowan
Sycamore
Large-leaved linden
Beech
Small-leaved lime
European aspen
Sweet chestnut
Norway spruce
Black locust
Days
150
100
50
0
1960
1980
2000
Year
Elevation (m a.s.l.)
1500
1000
500

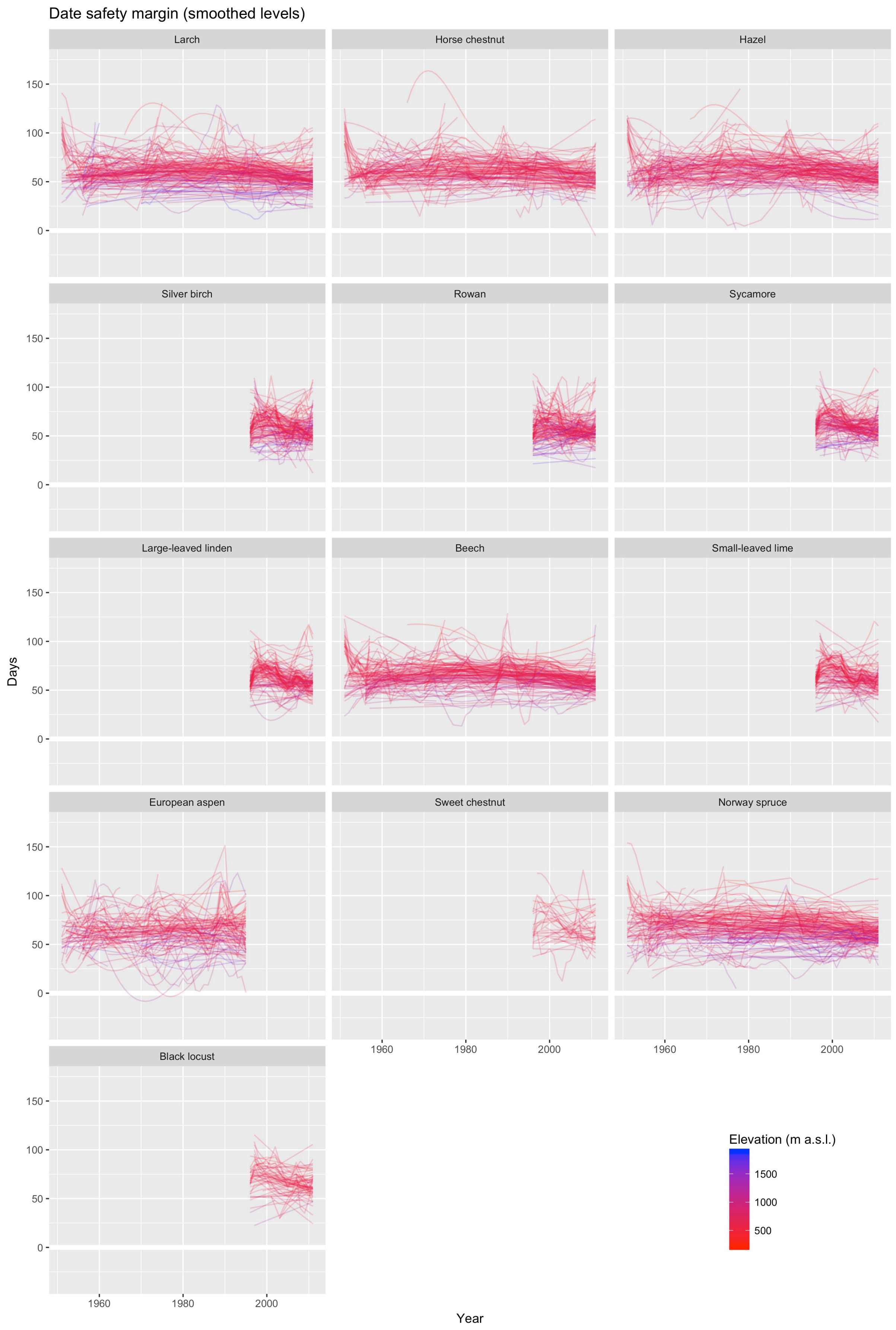
Date safety margin (smoothed levels)
Larch
Horse chestnut
Hazel
Silver birch
Rowan
Sycamore
Large-leaved linden
Beech
Small-leaved lime
European aspen
Sweet chestnut
Norway spruce
Black locust
Days
0
50
100
150
Year
1960
1980
2000
Elevation (m a.s.l.)
1500
1000
500

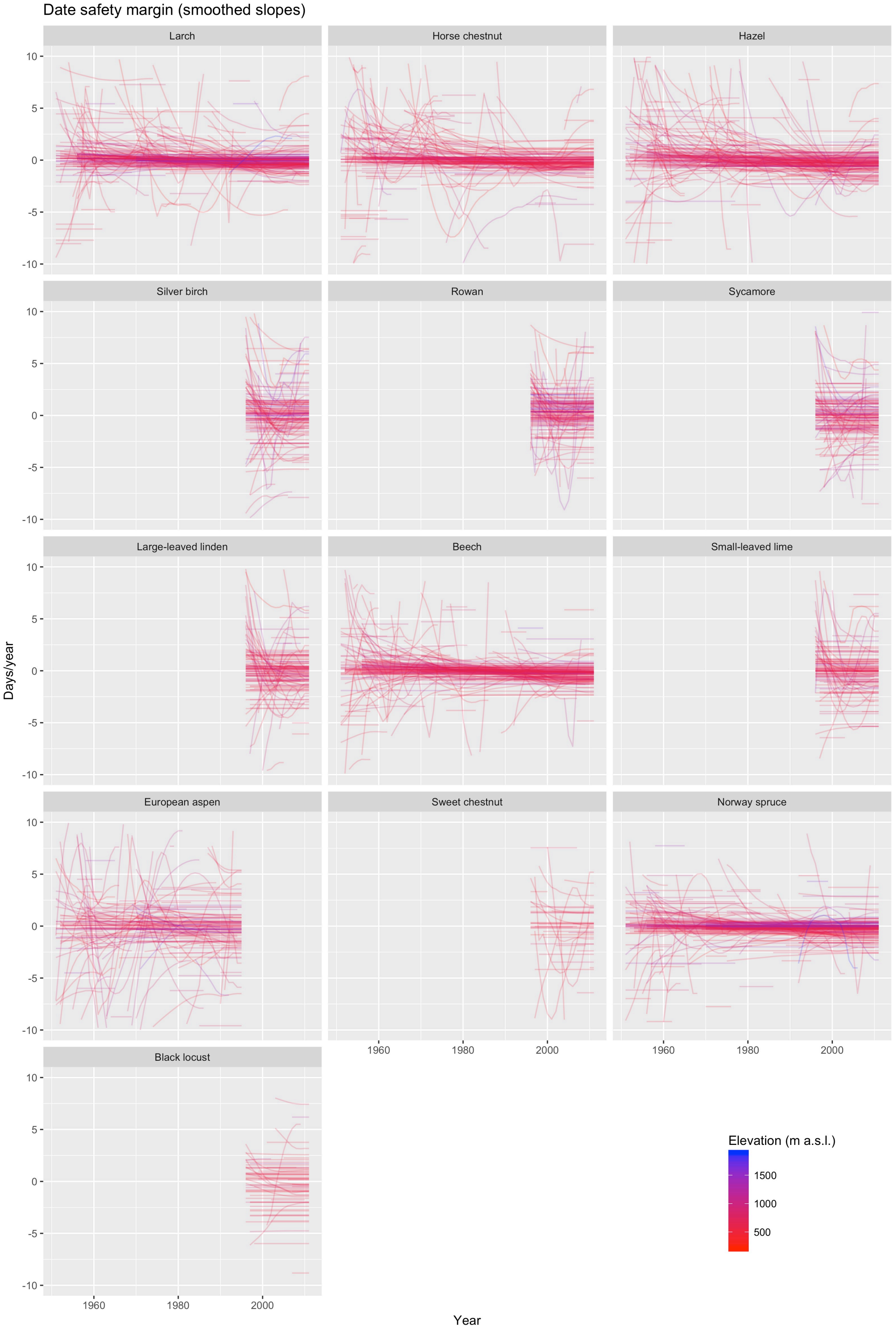
Date safety margin (smoothed slopes)
Larch
Horse chestnut
Hazel
Silver birch
Rowan
Sycamore
Large-leaved linden
Beech
Small-leaved lime
European aspen
Sweet chestnut
Norway spruce
Black locust
Days/year
10
5
0
-5
-10
1960
1980
2000
Year
Elevation (m a.s.l.)
1500
1000
500

Supplementary Figure S12 | Observations, smoothed levels and smoothed slopes of date safety margin. The smoothed levels and smoothed slopes are based on DLMs (see equations 5-8). The species are ordered according to median dates of leaf unfolding (see Fig. 3a). The color gradient represents the elevation of the stations (see Supplementary Table S1). For clarity, the y-axis was restricted to [-10, 10] for the smoothed slopes (< 0.87 % of data are omitted).

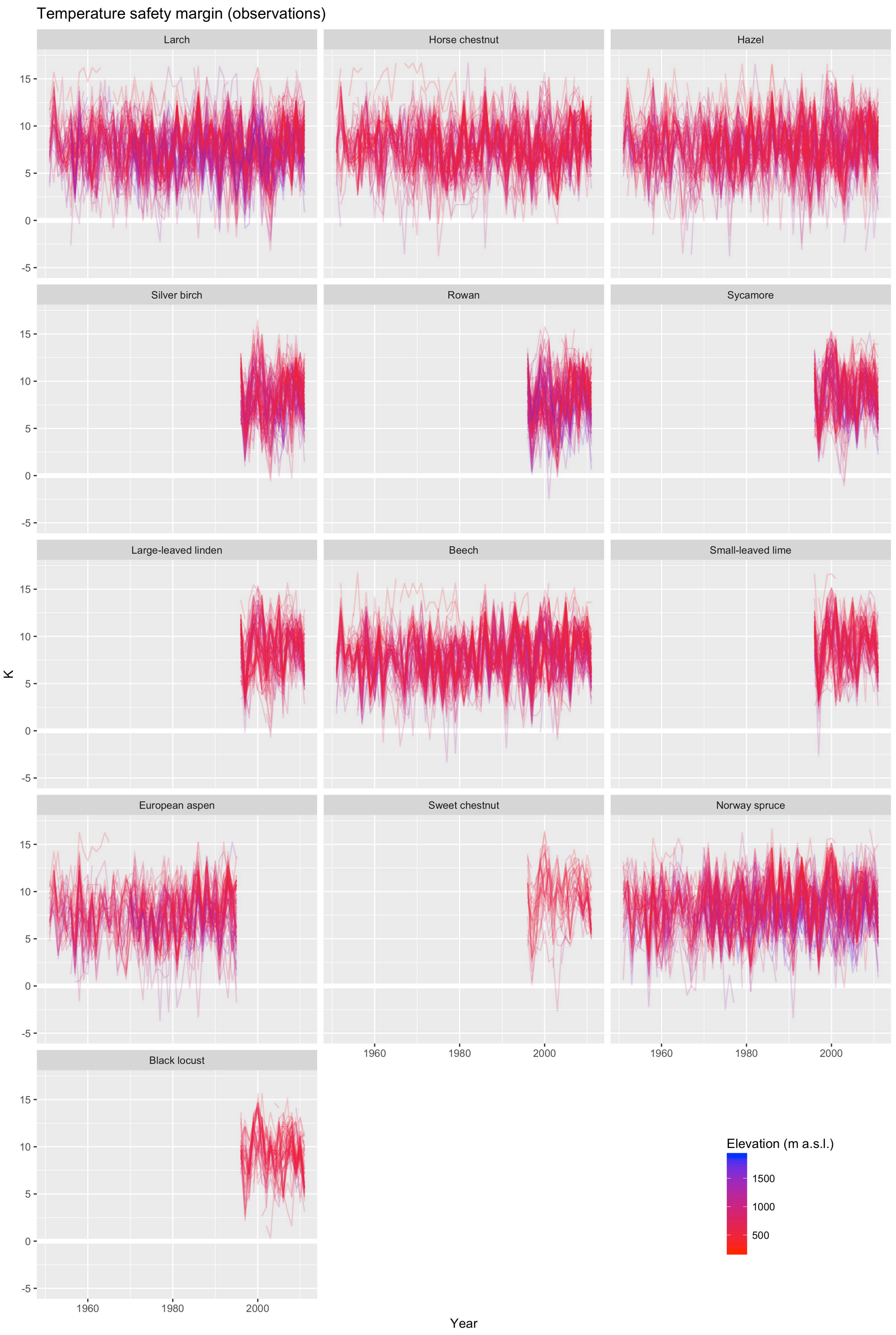

Temperature safety margin (observations)
Larch
Horse chestnut
Hazel
Silver birch
Rowan
Sycamore
Large-leaved linden
Beech
Small-leaved lime
European aspen
Sweet chestnut
Norway spruce
Black locust
K
15
10
5
0
-5
1960
1980
2000
Year
Elevation (m a.s.l.)
1500
1000
500

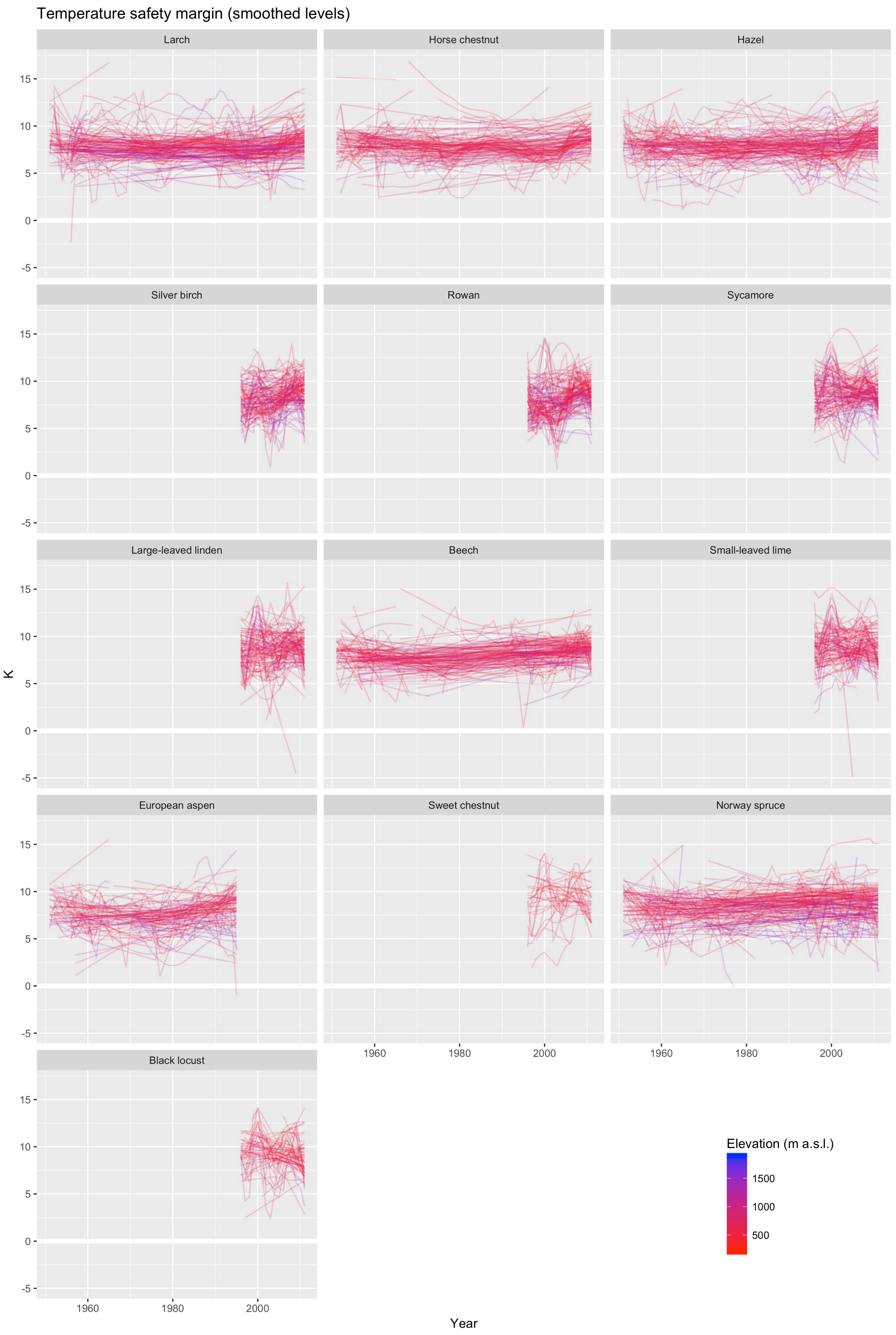

Temperature safety margin (smoothed levels)
Larch
Horse chestnut
Hazel
Silver birch
Rowan
Sycamore
Large-leaved linden
Beech
Small-leaved lime
European aspen
Sweet chestnut
Norway spruce
Black locust
K
15
10
5
0
-5
1960
1980
2000
Year
Elevation (m a.s.l.)
1500
1000
500

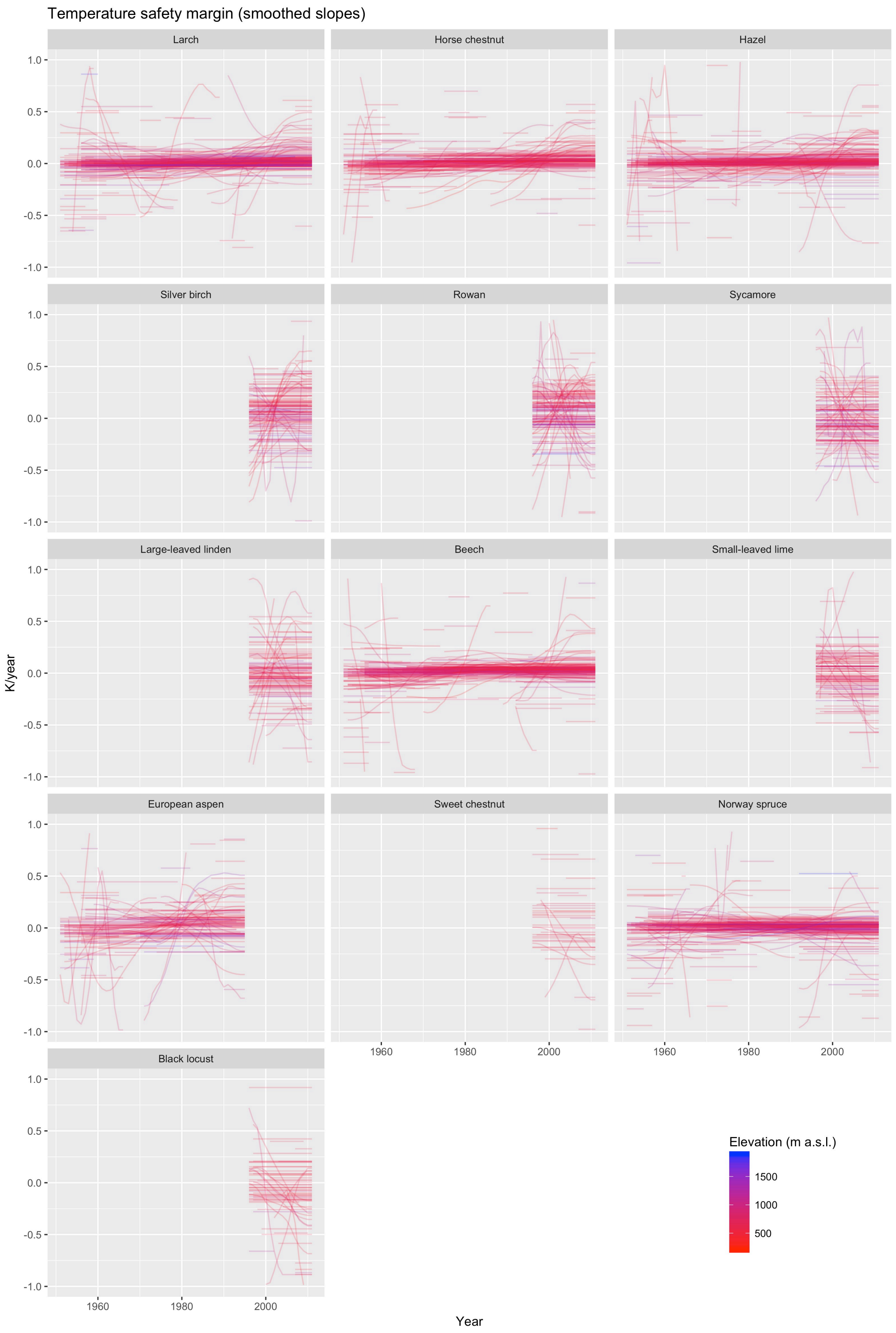
Temperature safety margin (smoothed slopes)
Larch
Horse chestnut
Hazel
Silver birch
Rowan
Sycamore
Large-leaved linden
Beech
Small-leaved lime
European aspen
Sweet chestnut
Norway spruce
Black locust
K/year
1.0
0.5
0.0
-0.5
-1.0
1960
1980
2000
Year
Elevation (m a.s.l.)
1500
1000
500

Supplementary Figure S13 | Observations, smoothed levels and smoothed slopes of temperature safety margin. The smoothed levels and smoothed slopes are based on DLMs (equations 5-8). The species are ordered according to median dates of leaf unfolding (see Fig. 3a). The color gradient represents the elevation of the stations (see Supplementary Table S1). For clarity, the y-axes were restricted to [-5, 17] for observations (< 0.016 % of data are omitted) and smoothed slopes (< 0.026 % omitted) and to [-1, 1] for smoothed slopes (< 0.50 % omitted).

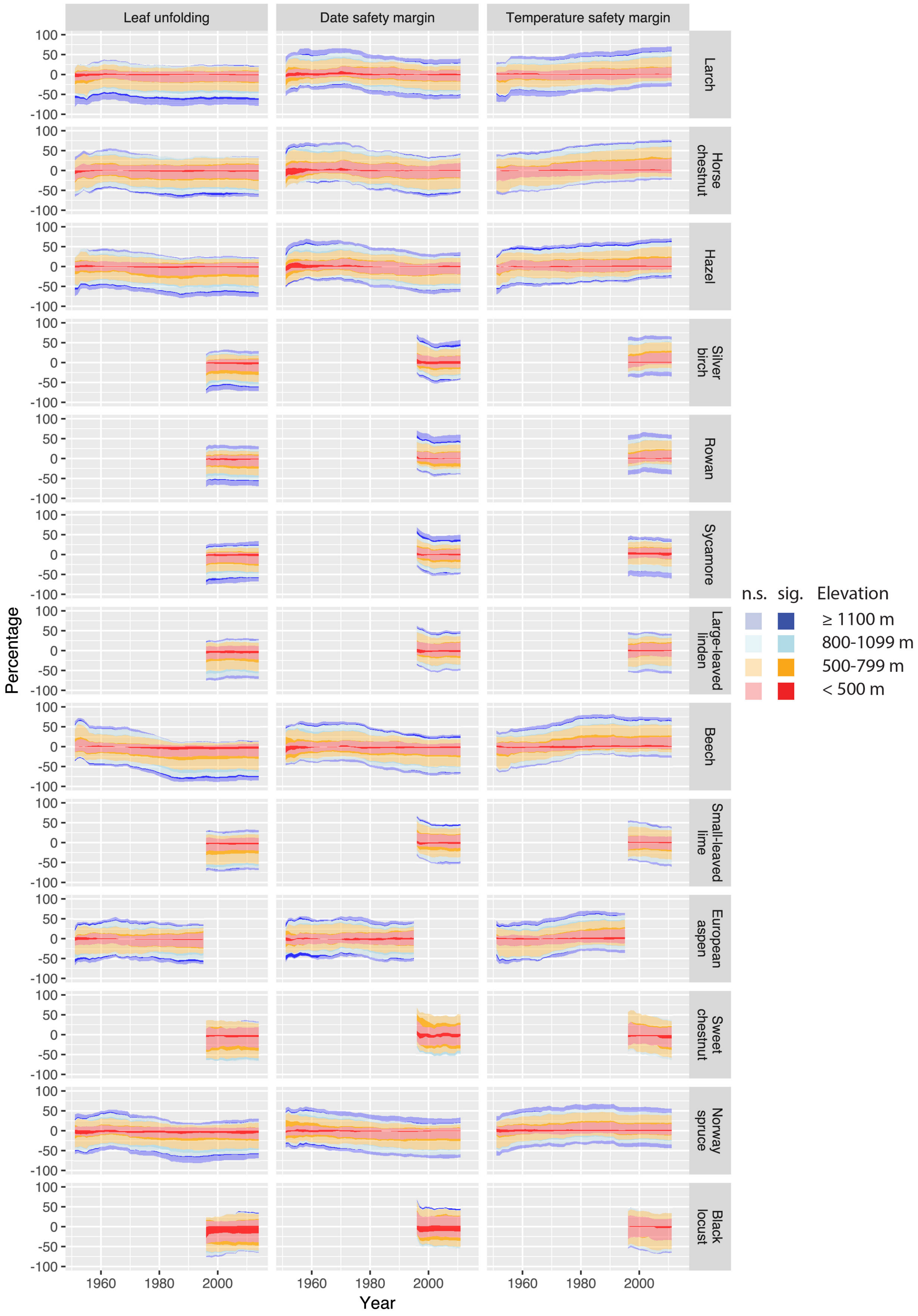

Leaf unfolding
Date safety margin
Temperature safety margin
Larch
Horse chestnut
Hazel
Silver birch
Rowan
Sycamore
Large-leaved linden
Beech
Small-leaved lime
European aspen
Sweet chestnut
Norway spruce
Black locust
Percentage
100
50
0
-50
-100
1960
1980
2000
Year
n.s.
sig.
Elevation
≥ 1100 m
800-1099 m
500-799 m
< 500 m

Supplementary Figure S14 | Shifts in leaf unfolding dates and frost risk (date safety margin and temperature safety margin) based on dynamic linear models (DLMs). Shown are percentages of stations assigned to four elevation bands with positive smoothed slopes (positive percentages on y-axis) and negative smoothed slopes (negative percentages on y-axis) based on DLMs (see equations 5-8). The species are ordered according to median dates of leaf unfolding (see Fig. 3a). All series from 1951 (1996 for species with later beginning of observations) to 2011 (2014 for leaf unfolding; 1995 for European aspen) from all stations were considered (number of series: larch, n=31-155; horse chestnut, n=31-125; hazel, n=35-145; silver birch, n=84-136; rowan, n=109-139; sycamore, n=88-135; large-leaved linden, n=84-120; beech, n=39-138; small-leaved lime, n=78-114; European aspen, n=24-99; sweet chestnut, n=19-43; Norway spruce, n=40-154; black locust, n=33-67). Non-significant slopes (n.s.) are shown with semi-transparent colors, significant slopes (sig.) with opaque colors. For each species and year, the absolute values of positive and negative percentages across all elevation bands sum up to 100 %. The plots do not substantially differ from those that consider complete series only (Fig. 4).

References

1. Lenz, A., Hoch, G., Körner, C. & Vitasse, Y. Convergence of leaf-out towards minimum risk of freezing damage in temperate trees. *Funct. Ecol.* **30,** 1480-1490 (2016).

2. Lenz, A., Hoch, G., Vitasse, Y. & Körner, C. European deciduous trees exhibit similar safety margins against damage by spring freeze events along elevational gradients. *New Phytol.* **200,** 1166-1175 (2013).

3. Vitasse, Y., Lenz, A., Hoch, G. & Körner, C. Earlier leaf-out rather than difference in freezing resistance puts juvenile trees at greater risk of damage than adult trees. *J. Ecol.* **102,** 981-988 (2014).

4. Taschler, D., Beikircher, B. & Neuner, G. Frost resistance and ice nucleation in leaves of five woody timberline species measured in situ during shoot expansion. *Tree Physiol.* **24,** 331-337 (2004).

5. Martin, M., Gavazov, K., Körner, C., Hättenschwiler, S. & Rixen, C. Reduced early growing season freezing resistance in alpine treeline plants under elevated atmospheric CO2. *Glob. Change Biol.* **16,** 1057-1070 (2010).

6. Repo, T. Seasonal changes of frost hardiness in Picea abies and Pinus sylvestris in Finland. *Can. J. Forest Res.* **22,** 1949-1957 (1992).
